# Supplementary material for: Distribution Analysis of the Lifespan Trait in Drosophila
Source: Int J Mol Sci. 2025 Dec 12;26(24):11987. doi: 10.3390/ijms262411987 (PMC12733365; doi:10.3390/ijms262411987)
Supplement: Supplementary file 1 [file ijms-26-11987-s001.zip › SUPPLI~1.DOC.pdf]

Supplementary S1

Main text. Additional sections

Supplementary to section 2.1 of the results

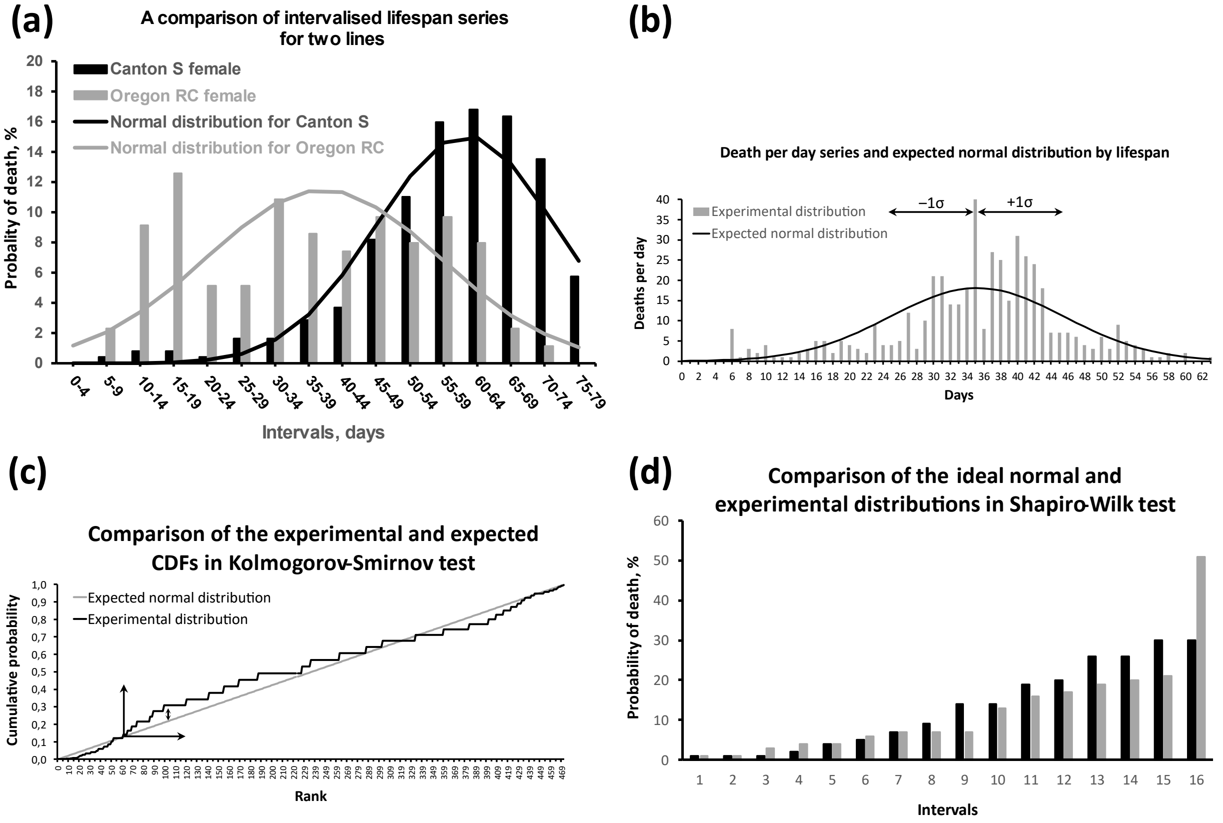

Figure S1. Distribution of lifespan with different interval sizes and the working principles of the Shapiro–Wilk (SW) and Kolmogorov–Smirnov (KS) tests. **(a)** A plot showing the frequency distribution of phenotypes by lifespan, as in Fig. 1c, but created using MS Excel. The black/gray curves show the normal distribution, which was calculated as described in the caption of Fig. 1a. **(b)** Data on the number of deaths per day of Oregon RC males (experiment with allele w^{1118} 2024) are presented. Black horizontal arrows indicate the ±1σ (standard deviation) limits. The black arc shows the normal distribution. **(c,d)** The working principles of the KS (C) and SW (D) tests for analysing single distributions are presented. **(c)** The line shows the cumulative expected normal distribution function (CDF) at $n \rightarrow \infty$, which is obtained as follows: Taking the inverse normal distribution function from the Rank/N value (see step-by-step instructions for performing the KS test in Appendix #2, Step 6) gives us the normal distribution function for the ranked expected sample, which increases gradually from 1 to n in increments of 1. The black curve shows the experimental CDF obtained by applying the normal distribution function with the μ and σ values of the sample to the experimental lifespan series. The data used to construct the graph for the experimental distribution are the same as in (A). The X-axis shows ranks (0, 1, 2, 3, etc.) and the Y-axis shows mortality/survival probabilities. The change in function should be understood as follows: If the curve deviates upwards, there is a succession of days with no mortality. If the curve deviates to the right, it indicates the presence of mortality, with the length of the step reflecting the number of individuals who died on a single day. The bidirectional arrow indicates the point at which the supremum of the function is observed, i.e. the location of the maximum difference between the experimental and expected CDFs. **(d)** The histogram shows a comparison of the reference ascending-sorted normal distribution (black bars) and the experimental ascending-sorted distribution (grey bars), which are superimposed on each other. Both distributions have 16 intervals. The reference distribution is obtained using the inverse CDF for an ideal (normal) lifespan series (Yumuhova et al., in preparation). The reference distribution has W = 1. The first and last days of mortality registration and the sample size (N) in the experimental and ideal normal series are identical. The data used to construct the graph for the experimental distribution are the same as in (A).

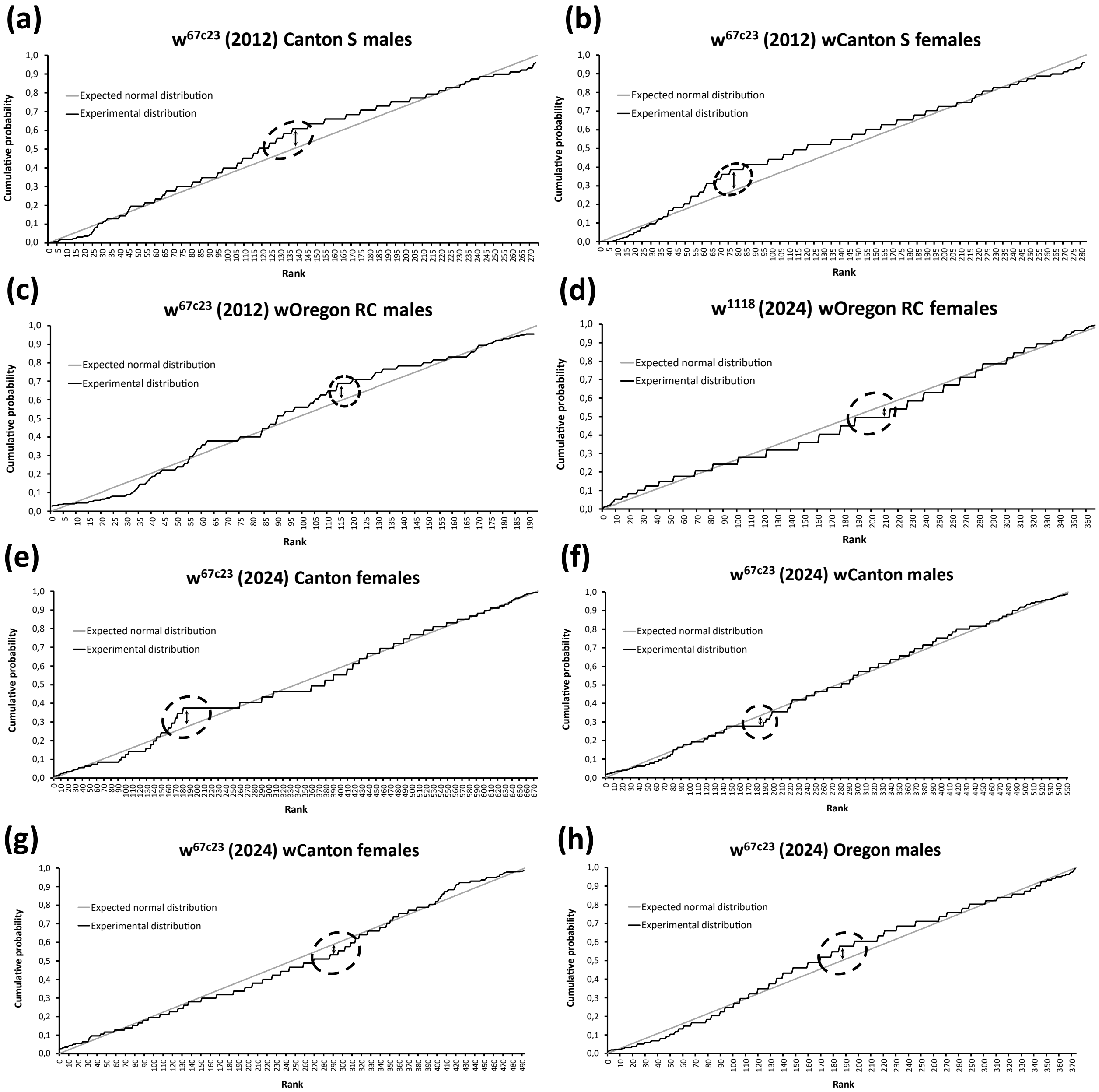

Figure S2. (**a-h**) Various cases of violation of normal distribution when using the Kolmogorov-Smirnov criterion are presented. The maximum deviation of the supremum of the Komogorov-Smirnov function from the rank line for the samples highlighted in bold in columns 8 and 10 in Table 1, 2, 3. All designations are as in Fig. S1c.

Supplementary to section 2.2 of the results

Additional observations on the mechanics of the KS test

1) The normal distribution function changes most intensely in the middle segment, and less intensely at the initial and final segments. Therefore, we can assume that the Kolmogorov–Smirnov test (KS test) result will be most strongly affected by fluctuations in mortality within the ±1σ interval in the middle of the distribution. To verify this, we performed additional tests.

It turned out that the same outlier in the middle and at the beginning/end of the lifespan series affects the KS statistic (D_n) differently. An outlier in the middle of the distribution causes a smaller increase in D_n than an outlier at the edges (Fig. S3, Table S1). This leads to an increased probability of defining the distribution as normal in

situations where the outlier is in the middle of the distribution. Therefore, the KS test is sensitive to the location of outliers in the lifespan series.

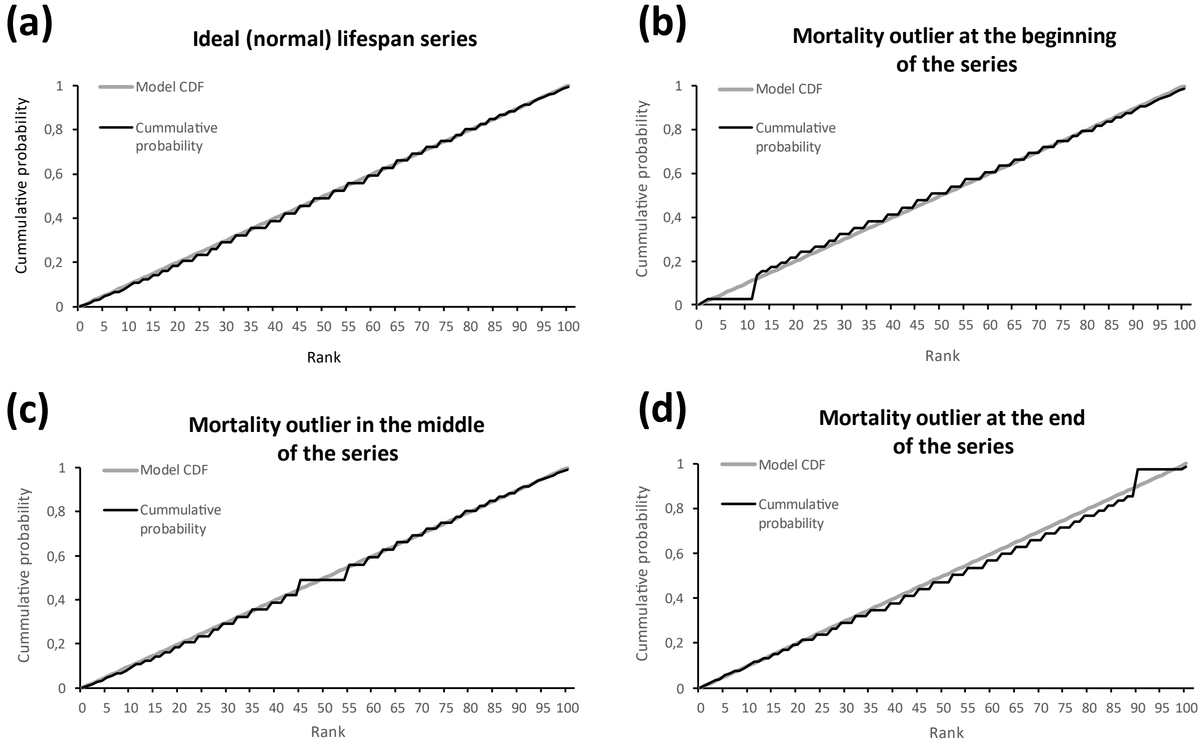

Figure S3. Peculiarities of KS test performance in the presence of mortality outliers in the initial (**b**), median (**c**) and end (**d**) sections of the ideal lifespan series (**a**). For these experiments, we used an ideal normal lifespan series (see Figure S5). The size of the outlier was 10 individuals at N = 100.

Table S1. Changes in KS test statistic and p-value in response to the same mortality outlier at different parts of the lifespan series.

Ideal (normal) lifespan series	μ	32.28	D_n	0.025
	σ	11.44	p-value	1
Mortality outlier at the beginning of the series	μ	31.71	D_n	0.081
	σ	12.41	p-value	0.522
Mortality outlier in the middle of the series	μ	32.28	D_n	0.050
	σ	11.43	p-value	0.772
Mortality outlier at the end of the series	μ	32.88	D_n	0.083
	σ	12.47	p-value	0.498

2) Additionally, we found that the KS test result was significantly dependent on the sample size. When the sample size was doubled, the value of the test statistic Dn remained at 0.080 for both the original sample (N = 209) and the doubled sample (N = 418) (Figure S4). This indicates that the supremum point and the difference between the experimental and control CDFs remained unchanged. However, as the $\sqrt{N}$ value increased, the D-critical and p-value decreased from D_{crit} = 0.0939 (α = 0.05) and P = 0.1269 (normal distribution) for the original sample to D_{crit} = 0.0664 (α = 0.05) and P = 0.0080 (non-normal distribution) for the doubled sample. Therefore, as the sample size increased, the KS test incorrectly characterised an initially normally distributed sample as non-normally distributed. This effect on the results of the KS test is a consequence of the specificity of survival data. Specifically, since the maximum lifespan of individuals in the sample is limited and cannot rise with an increase in sample size, the number of repeated values in the lifespan series rises as the sample size increases. Consequently, the length of the steps of the function increases (the function shifts to the right) and the probability of defining the distribution as non-normal increases. Thus, the KS test will always tend to classify small samples as normally distributed and large samples as non-normally

distributed; the smaller the sample size, the more likely the distribution is to be normal. This feature of the KS test is most evident when the sample is partitioned into intervals (i.e. in the absence of Lilliefors correction). In this case, the number of observation points was small (12–17), and a normal distribution was detected for all cases studied (Tables 1, 2 and 3, column 12).

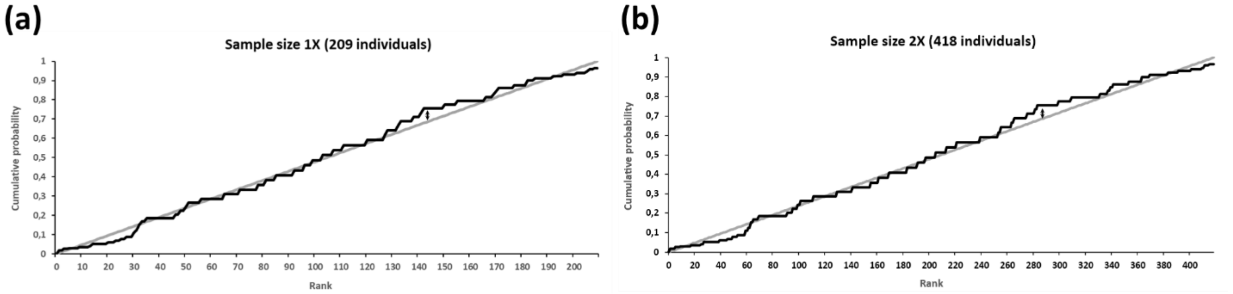

Figure S4. Dependence of KS test results on sample size. (**a**) sample size: 209 individuals. (**b**) sample size: 418 individuals. The black arrow indicates the supremum of the Kolmogorov function. The lifespan series of w^{67c23} Oregon RC female flies (experiment with the w^{67c23} allele, 2012 was used for plotting.

Supplementary to section 2.3 of the results

Ideal (normal) lifespan series
with n=100, begin=5, end=50

5, 8, 9, 11, 12, 13, 13, 14, 15, 15, 16, 16, 17, 17, 18, 18, 18, 19, 19, 19,
20, 20, 20, 21, 21, 21, 22, 22, 22, 23, 23, 23, 23, 24, 24, 24, 24, 25, 25, 25,
25, 26, 26, 26, 26, 27, 27, 27, 27, 28, 28, 28, 28, 29, 29, 29, 29, 30, 30, 30,
30, 31, 31, 31, 31, 32, 32, 32, 32, 33, 33, 33, 34, 34, 34, 34, 35, 35, 35, 36,
36, 36, 37, 37, 38, 38, 38, 39, 39, 40, 40, 41, 42, 42, 43, 44, 45, 46, 48, 50.

Figure S5. This figure shows an example of a lifespan series (*N* = 100 individuals) forming an ideal (normal) lifespan distribution. The initial and final days of mortality recording are the '5th' and '50th' days, respectively.

Table S2. Reproducibility of the Shapiro–Wilk test depending on the initial day of intervalization on an ideal normal lifespan series.

1	2	3	4			5			6			7			8		
Genotype	Sex	μ, days	Internalization* from														
			day 0			day 1			day 2			day 3			day 4		
			W	p-value **	Number of intervals	W	p-value	Number of intervals	W	p-value	Number of intervals	W	p-value	Number of intervals	W	p-value	Number of intervals
w^{1118} (2024)																	
Canton S	M	36.75	0.89	0.098	13	0.88	0.055	14	0.87	0.047	14	0.88	0.056	14	0.90	0.114	13
	F	37.26	0.89	0.105	13	0.88	0.064	14	0.89	0.073	14	0.88	0.064	14	0.88	0.065	14
w^{1118} Canton S	M	35.23	0.90	0.097	14	0.89	0.074	14	0.89	0.079	14	0.89	0.110	13	0.90	0.138	13
	F	36.25	0.90	0.131	13	0.90	0.130	13	0.90	0.147	13	0.90	0.123	13	0.90	0.130	13
Oregon RC	M	34.71	0.87	0.065	12	0.88	0.093	12	0.87	0.060	13	0.87	0.051	13	0.89	0.113	12
	F	33.23	0.90	0.178	11	0.89	0.130	11	0.90	0.175	11	0.87	0.069	12	0.86	0.045	12
w^{1118} Oregon RC	M	29.76	0.87	0.068	12	0.89	0.108	12	0.89	0.115	12	0.89	0.145	11	0.91	0.249	11
	F	26.23	0.89	0.126	11	0.89	0.167	10	0.88	0.147	10	0.90	0.216	10	0.88	0.148	10
w^{67c23} (2024)																	
Canton S	M	36.98	0.87	0.031	15	0.88	0.045	15	0.87	0.037	15	0.88	0.040	15	0.89	0.070	14
	F	33.08	0.87	0.045	14	0.88	0.068	13	0.89	0.094	13	0.88	0.082	13	0.89	0.086	13
w^{67c23} Canton S	M	41.21	0.88	0.029	17	0.88	0.028	17	0.88	0.043	16	0.89	0.047	16	0.89	0.050	16
	F	39.75	0.87	0.031	16	0.88	0.043	16	0.89	0.050	16	0.88	0.042	16	0.90	0.082	15
Oregon RC	M	36.75	0.88	0.041	15	0.89	0.091	14	0.88	0.064	14	0.89	0.069	14	0.89	0.092	14
	F	32.26	0.87	0.051	13	0.89	0.123	12	0.88	0.084	12	0.87	0.069	12	0.89	0.127	12
w^{67c23} Oregon RC	M	41.12	0.88	0.028	17	0.88	0.027	17	0.88	0.036	16	0.88	0.046	16	0.89	0.047	16
	F	36.59	0.87	0.039	15	0.88	0.045	15	0.87	0.033	15	0.89	0.072	14	0.89	0.071	14
w^{67c23} (2012)																	
Canton S	M	39.74	0.89	0.078	14	0.88	0.050	15	0.89	0.069	15	0.89	0.063	15	0.89	0.065	15
	F	43.75	0.89	0.070	15	0.90	0.097	15	0.90	0.081	15	0.90	0.098	15	0.87	0.031	16

w^{67c23} Canton S	M	43.75	0.90	0.070	16	0.89	0.038	17	0.88	0.032	17	0.90	0.068	16	0.89	0.056	16
	F	42.24	0.88	0.047	15	0.90	0.099	15	0.88	0.041	16	0.88	0.037	16	0.90	0.112	15
Oregon RC	M	40.26	0.89	0.075	15	0.89	0.080	15	0.89	0.073	15	0.89	0.069	15	0.90	0.103	15
	F	37.75	0.88	0.068	14	0.89	0.077	14	0.90	0.109	14	0.90	0.131	14	0.89	0.070	14
w^{67c23} Oregon RC	M	37.73	0.91	0.166	13	0.90	0.125	13	0.89	0.095	14	0.89	0.078	14	0.90	0.096	14
	F	37.75	0.90	0.127	13	0.89	0.102	13	0.90	0.121	13	0.90	0.140	12	0.90	0.120	13

* Interval size: 5 days.

** With a significance threshold of $P \geq 0.05$, the distribution was considered normal.

Supplementary to section 2.4 of the results

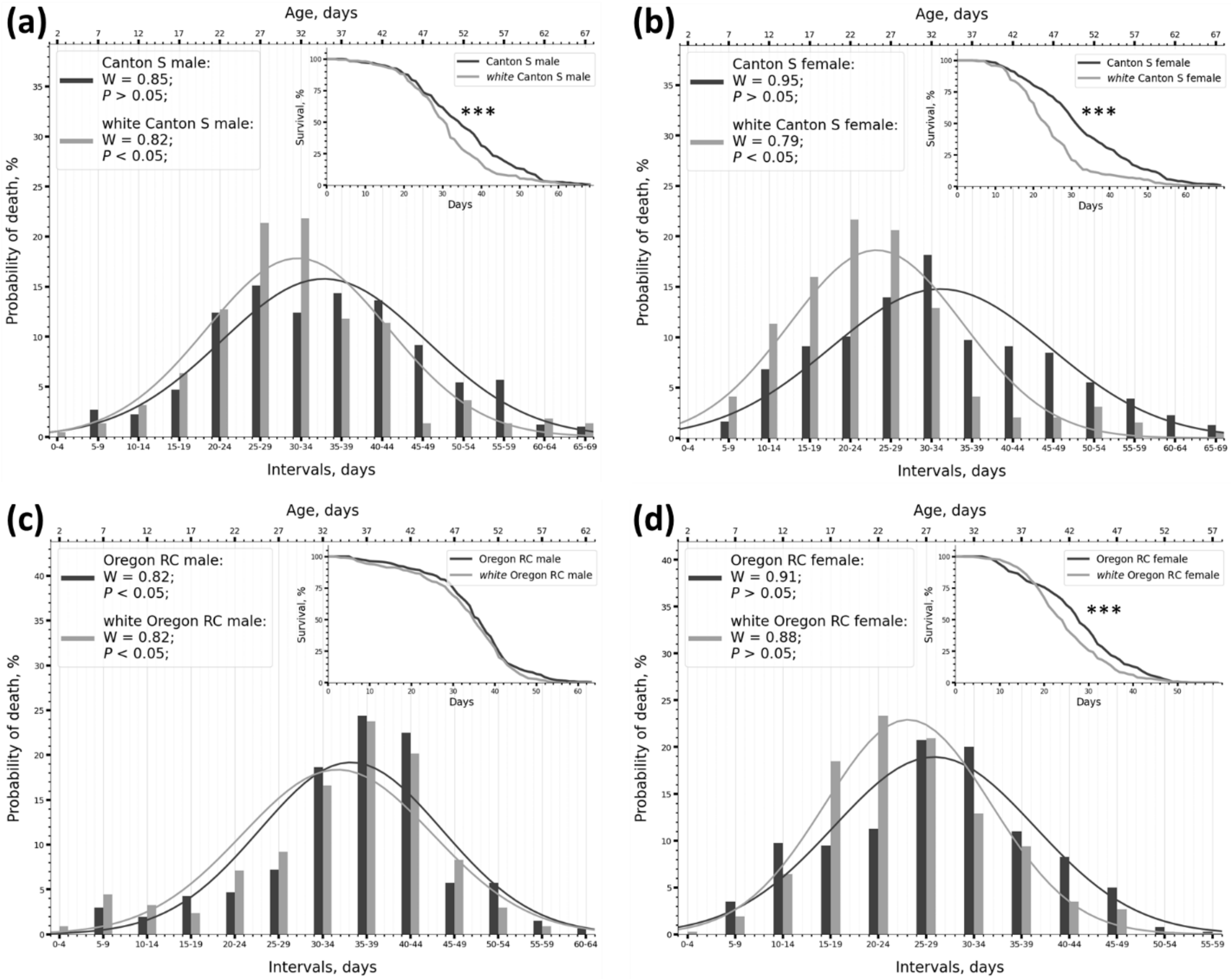

Figure S6. Analysis of data from the experiment with allele w^{1118} 2024 is presented. (**a, b**) Canton S genetic background; (**c, d**) Oregon RC genetic background; (**a, c**) Comparison of WT males with *white* mutant males; (**b, d**) Comparisons of WT females with *white* mutant females. Phenotype frequencies/probabilities by lifespan (bars) are shown with superimposed normal distributions. Frequency distributions were obtained by dividing the original lifespan series into 5-day intervals. W is the Shapiro–Wilk (SW) test statistic and *P* is the SW test p-value. The inset in the upper right corner shows the survival curves for the compared samples. The genotypes are indicated in the legend. ****P* < 0.001.

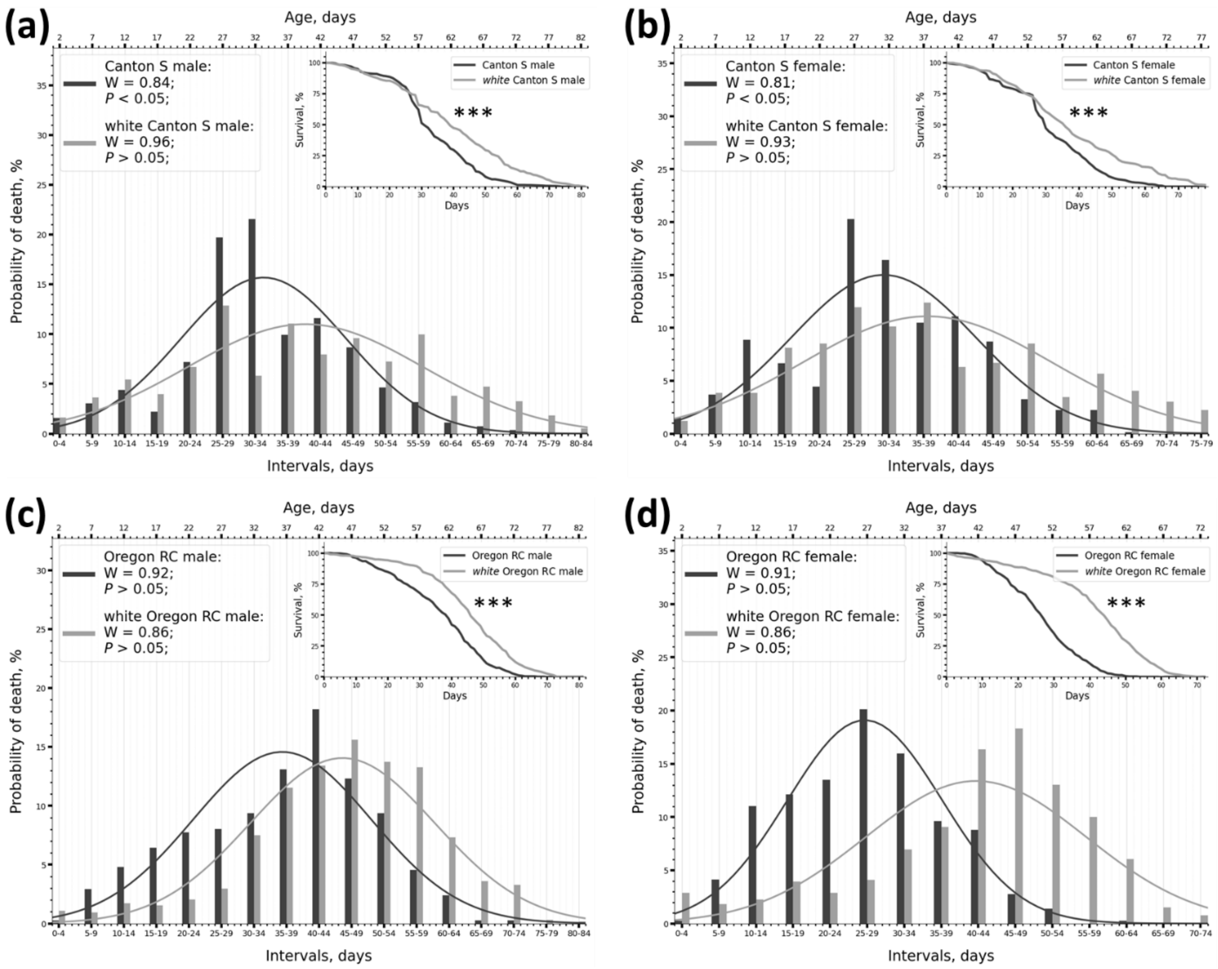

Figure S7. Analysis of data from the experiment with allele w^{67c23} 2024 is presented. All designations as in Fig. 1c and Fig. S6.

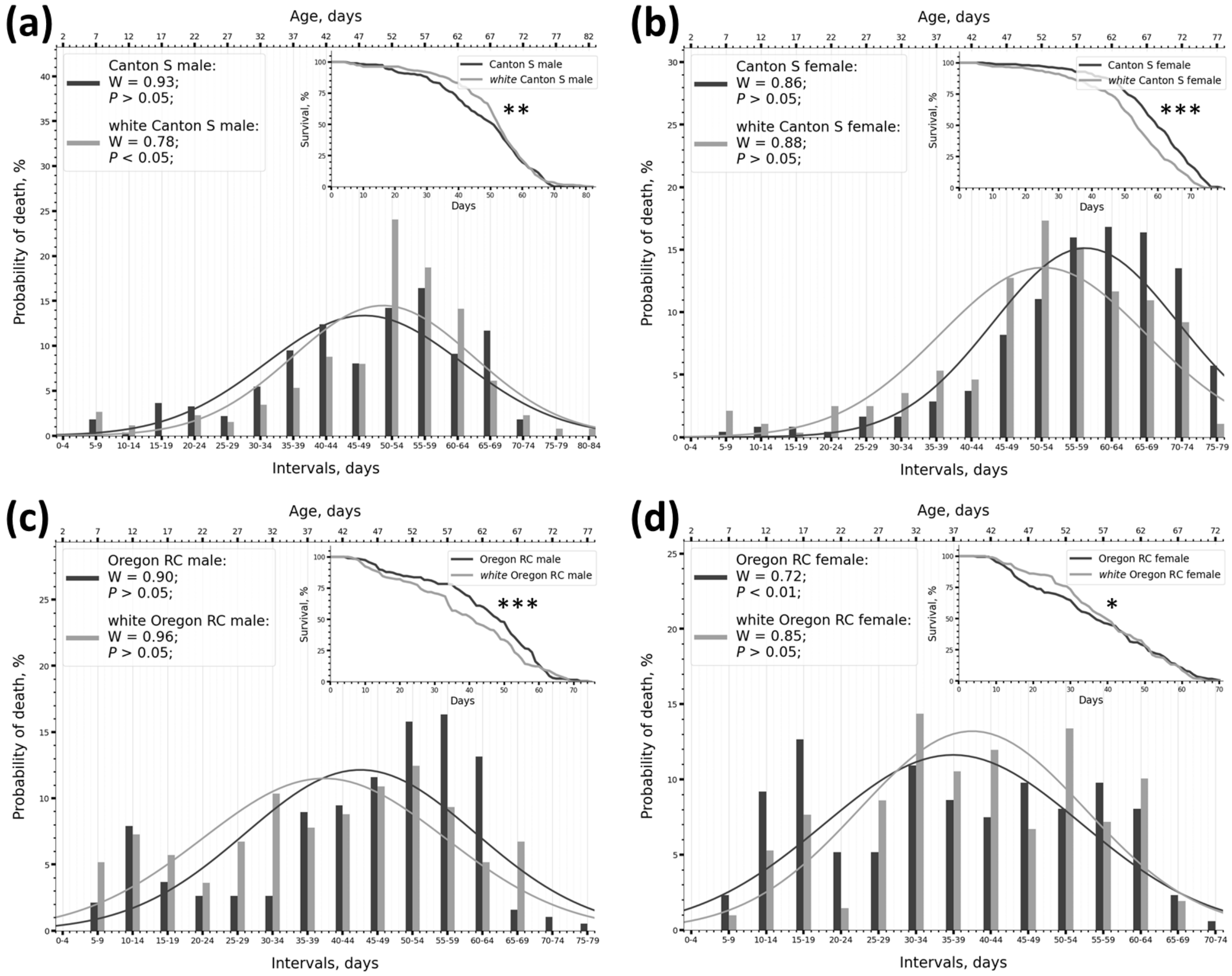

Figure S8. Analysis of data from the experiment with allele w^{67c23} 2012 is presented. All designations as in Fig. 1c and Fig. S6. $**P < 0.01$. $*P < 0.05$.

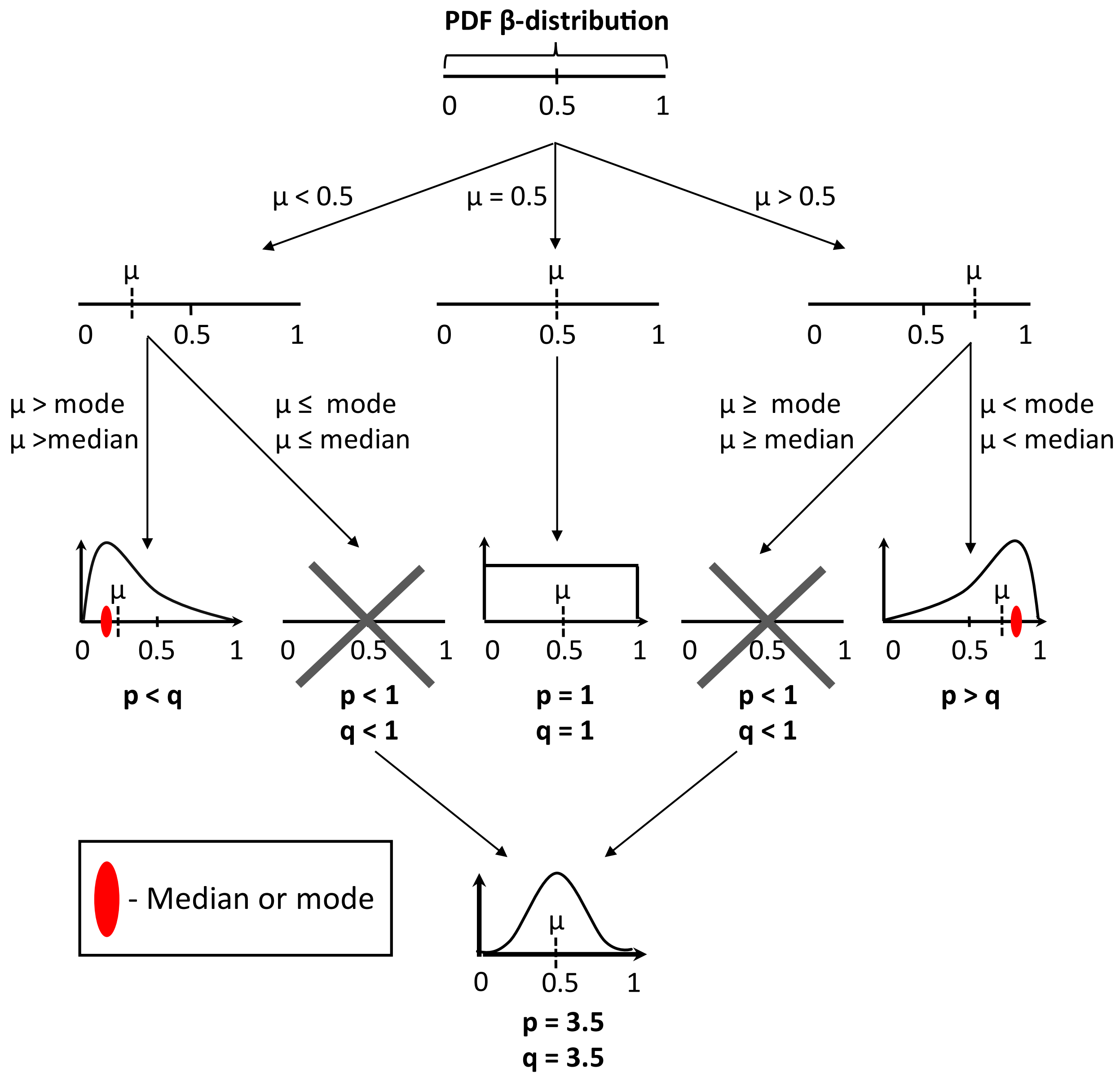

Figure S9. Decision tree for *β*-distribution. This shows the paths and criteria for selecting the form of the *β*-distribution that best describes the frequency distribution of phenotypes by lifespan. If a left- or right-skewed β-distribution is not possible, a normal (bell-shaped) distribution with *p* and *q* values of 3.5 is selected. If *μ* is close to 0.5 (see section 3.7 of Materials and Methods for details of how to calculate this), and *p* and *q* are close to 1, a distribution similar to a plateau-shaped (uniform) distribution is selected.

Supplementary to the discussion section

Table S3. A comparison of the effectiveness of Kolmogorov's and Marsaglia's formulas for calculating p-values using 24 experimental samples.

	Kolmogorov's formula on data w/o intervals, p-value	Marsaglia's formula on data w/o intervals, p-value	Kolmogorov's formula on data with intervals, p-value	Marsaglia's formula on data with intervals, p-value
Canton S Male (2024)	0.285	0.280	0.646	0.602
Canton S Female (2024)	0.091	0.088	0.917	1.014
w^{1118} Canton S Male (2024)	0.018	0.017	0.307	0.258
w^{1118} Canton S Female (2024)	0.011	0.010	0.197	0.156

Oregon R Male (2024)	< 0.001	< 0.001	0.212	0.168
Oregon R Female (2024)	0.115	0.111	0.834	0.845
w^{1118} Oregon R Male (2024)	0.001	0.001	0.702	0.664
w^{1118} Oregon R Female (2024)	0.019	0.018	0.725	0.689
Canton S Male (2024)	< 0.001	< 0.001	0.443	0.391
Canton S Female (2024)	< 0.001	< 0.001	0.740	0.721
w^{67c23} Canton S Male (2024)	0.020	0.019	0.960	1.148
w^{67c23} Canton S Female (2024)	0.012	0.012	0.654	0.621
Oregon R Male (2024)	0.013	0.013	0.994	1.354
Oregon R Female (2024)	0.254	0.249	0.839	0.863
w^{67c23} Oregon R Male (2024)	0.019	0.018	0.222	0.186
w^{67c23} Oregon R Female (2024)	< 0.001	< 0.001	0.479	0.428
Canton S Male (2012)	0.003	0.003	0.720	0.696
Canton S Female (2012)	0.046	0.044	0.473	0.422
w^{67c23} Canton S Male (2012)	0.001	< 0.001	0.406	0.358
w^{67c23} Canton S Female (2012)	0.001	0.001	0.594	0.549
Oregon R Male (2012)	0.003	0.003	0.332	0.284
Oregon R Female (2012)	0.098	0.093	0.773	0.765
w^{67c23} Oregon R Male (2012)	0.043	0.040	0.996	1.391
w^{67c23} Oregon R Female (2012)	0.127	0.122	0.951	1.106

Supplementary S2

Calculation of the one sample Kolmogorov–Smirnov normality test for lifespan data without partitioning into intervals. Step-by-step instruction.

Introductory notes

Here, we present the procedure for calculating the one-sample Kolmogorov-Smirnov (KS) test on lifespan series without dividing them into intervals. Our calculations have shown that when the KS test is applied to intervalized lifespan series (12-18 intervals), the results always indicate a normal distribution (see the main text). However, the Shapiro-Wilk test shows that some of these data are not normal.

Step 1. Convert the deaths per day series into a lifespan series. This conversion can be done using an algorithm below.

If the deaths per day series has zero deaths on a given day, we do not add any number to the lifespan series. If the deaths per day series has non-zero deaths on a given day, we add the number of days on which non-zero deaths occurred as many times as the number of deaths that occurred on that day.
Paste the lifespan series, which is a row in which each number represents the age in days at which an individual from the sample died. Sort this row from lowest to highest.

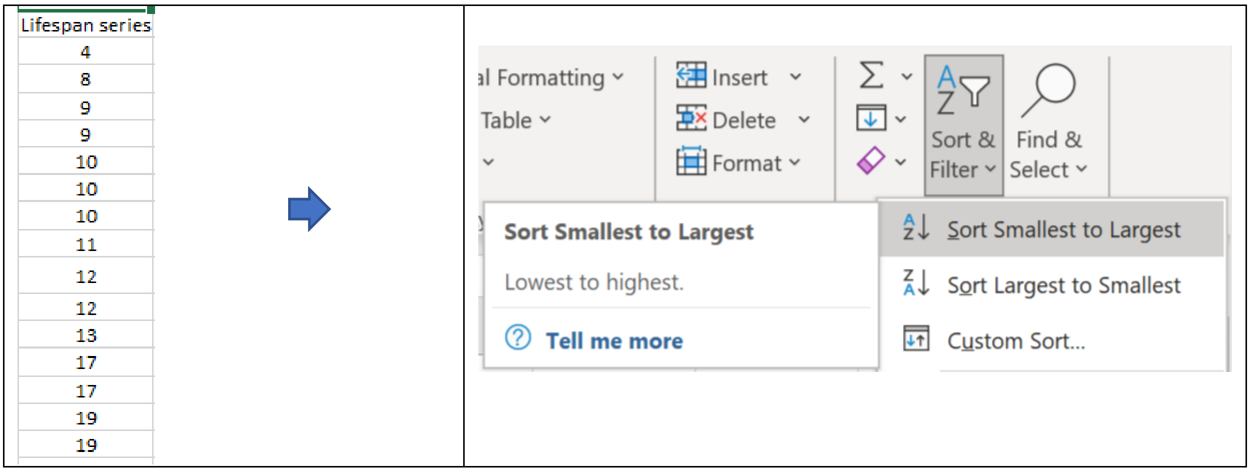

Step 2. Assign a rank to each number.

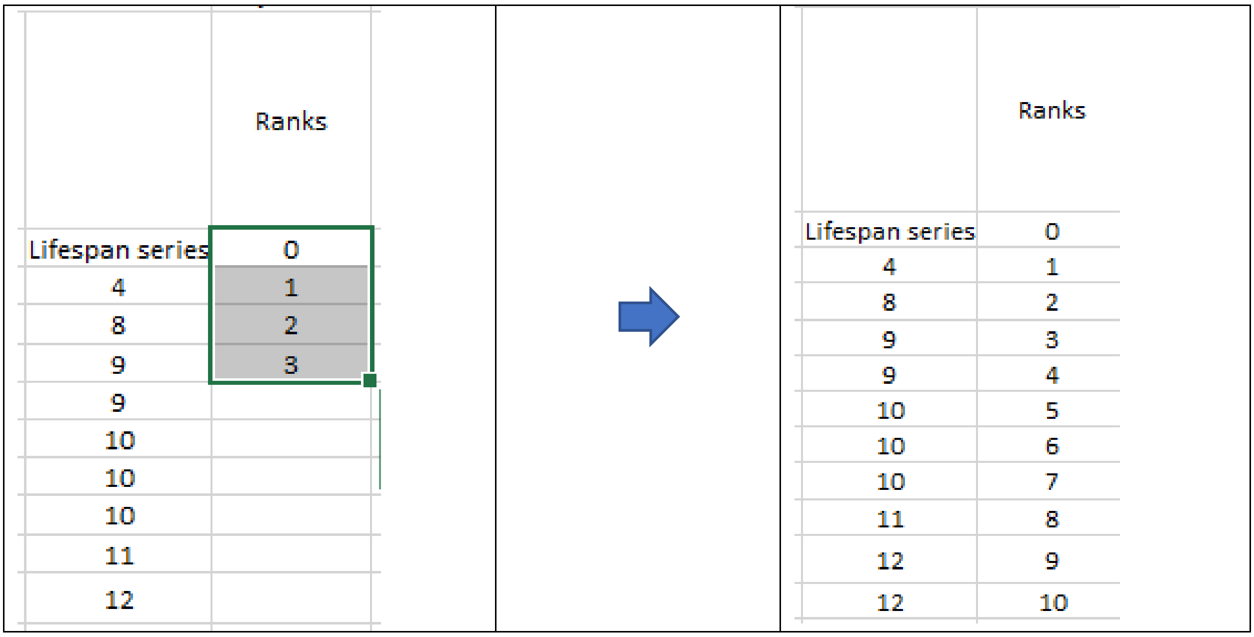

Step 3. Determine the sample size (*N*). There are three ways to do this (Table below):

1) Use the SUM function to add up all the daily death values in column 1.
2) Use the COUNT function to count the number of cells in the lifespan series (column 3).
3) Take the highest rank in column 4.

1)

			Ranks	N
Days	Deaths per day	Lifespan series	0	
1	0	4	1	=SUM(B9:B84)
2	0	8	2	or
3	0	9	3	197
4	1	9	4	or
5	0	10	5	197
6	0	10	6	
7	0	10	7	
8	1	11	8	

2)

			Ranks	N
Days	Deaths per day	Lifespan series	0	
1	0	4	1	197
2	0	8	2	or
3	0	9	3	=COUNT(C9:C205)
4	1	9	4	or
5	0	10	5	197
6	0	10	6	
7	0	10	7	
8	1	11	8	

3)

			Ranks	N
Days	Deaths per day	Lifespan series	0	
1	0	4	1	197
2	0	8	2	or
3	0	9	3	197
4	1	9	4	or
5	0	10	5	=D205
6	0	10	6	
		76	197	

Step 4. Use the AVERAGE function to calculate the mean lifespan (μ).

	Step 2	Step 3	Step 4
	Ranks	N	Mean (μ)
Lifespan series	0		
4	1	197	=AVERAGE(C9:C205)
8	2	or	
9	3	197	
9	4	or	
10	5	197	

Step 5. Use the STDEV.S function to calculate the standard deviation (SD, σ).

	Step 2	Step 3	Step 4	Step 5
	Ranks	N	Mean (μ)	Standard deviation
Lifespan series	0			
4	1	197	48,34	=STDEV.S(C9:C205)
8	2	or		
9	3	197		
9	4	or		
10	5	197		
10	6			
10	7			
11	8			

Step 6. Create the model/expected/theoretical cumulative normal distribution function (CDF) using the rank row (*i*/*N*, where *i* is the rank).

Ranks	N	Model CDF from the right
0		Rank/N
1	197	=D7/$E$7
2	or	
3	197	
4	or	
5	197	
6		
7		

Ranks	N	Model CDF from the right
0		Rank/N
1	197	0,0050761
2	or	
3	197	
4	or	
5	197	
6		
7		

Ranks	N	Model CDF from the right
0		Rank/N
1	197	0,0050761
2	or	0,0101523
3	197	0,0152284
4	or	0,0203046
5	197	0,0253807
6		0,0304569
7		0,035533

Step 7. Create the adjusted model/expected/theoretical cumulative normal distribution function (CDF) using the rank row (*i*-1/*N*, where *i* is the rank and *i*-1 is the adjustment).

Ranks	N	Model CDF from the right	Model CDF from the left
0		Rank/N	Rank-1/N
1	197	=(D7-1)/$E$7	
2	or	0,0101523	
3	197	0,0152284	
4	or	0,0203046	
5	197	0,0253807	
6		0,0304569	
7		0,035533	
8		0,0406091	
9		0,0456853	
10		0,0507614	
11		0,0558376	
12		0,0609137	
13		0,0659898	
14		0,071066	
15		0,0761421	

Ranks	N	Model CDF from the right	Model CDF from the left
0		Rank/N	Rank-1/N
1	197	0,0050761	0
2	or	0,0101523	
3	197	0,0152284	
4	or	0,0203046	
5	197	0,0253807	
6		0,0304569	
7		0,035533	
8		0,0406091	
9		0,0456853	
10		0,0507614	
11		0,0558376	
12		0,0609137	
13		0,0659898	
14		0,071066	
15		0,0761421	

Ranks	N	Model CDF from the right	Model CDF from the left
0		Rank/N	Rank-1/N
1	197	0,0050761	0
2	or	0,0101523	0,0050761
3	197	0,0152284	0,0101523
4	or	0,0203046	0,0152284
5	197	0,0253807	0,0203046
6		0,0304569	0,0253807
7		0,035533	0,0304569
8		0,0406091	0,035533
9		0,0456853	0,0406091
10		0,0507614	0,0456853
11		0,0558376	0,0507614
12		0,0609137	0,0558376
13		0,0659898	0,0609137
14		0,071066	0,0659898
15		0,0761421	0,071066

Step 8. Create the cumulative distribution function (CDF) from the lifespan data series using the NORM.DIST function (age of the fly; *μ*; σ; TRUE), where TRUE (1) is the cumulative density function and FALSE (0) is the probability density function.

LifeSpan data	Mean	Standard deviation	Model CDF from the left	Cummulative Normal Distribution Function (CDF)
♂ (M)			Rank-1/N	
4	48,34	16,90	=NORM.DIST(C7;$F$7;$G$7;TRUE)	
8			NORM.DIST(x; mean; standard_dev; **cumulative**)	
9			0,0101523	
9			0,0152284	
10			0,0203046	
10			0,0253807	
10			0,0304569	

LifeSpan data	Ranks	N	Mean	Standard deviation	Model CDF from the left	Cummulative Normal Distribution Function (CDF)
♂ (M)	0				Rank-1/N	
4	1	197	48,34	16,90	0	0,004339967
8	2	or			0,0050761	0,008478083
9	3	197			0,0101523	0,009943615
9	4	or			0,0152284	0,009943615
10	5	197			0,0203046	0,011625636
10	6				0,0253807	0,011625636
10	7				0,0304569	0,011625636
11	8				0,035533	0,013549377
12	9				0,0406091	0,015741882
12	10				0,0456853	0,015741882
13	11				0,0507614	0,018231964
17	12				0,0558376	0,031800733
17	13				0,0609137	0,031800733

Step 9. Use the ABS function to find the absolute differences between the theoretical (Rank/N) and experimental CDF.

Model CDF from the right	Cummulative Normal Distribution Function (CDF)	difference from model (+)
Rank/N		
0,005076142	0,004339967	=ABS(K7-I7)
0,010152284	0,008478083	ABS(number)
0,015228426	0,009943615	
0,020304569	0,009943615	
0,025380711	0,011625636	
0,030456853	0,011625636	
0,035532995	0,011625636	
0,040609137	0,013549377	
0,045685279	0,015741882	
0,050761421	0,015741882	
0,055837563	0,018231964	

Model CDF from the right	Cummulative Normal Distribution Function (CDF)	difference from model (+)
Rank/N		
0,005076142	0,004339967	0,000736175
0,010152284	0,008478083	0,001674202
0,015228426	0,009943615	0,005284811
0,020304569	0,009943615	0,010360953
0,025380711	0,011625636	0,013755074
0,030456853	0,011625636	0,018831216
0,035532995	0,011625636	0,023907359
0,040609137	0,013549377	0,02705976
0,045685279	0,015741882	0,029943397
0,050761421	0,015741882	0,035019539
0,055837563	0,018231964	0,037605599

Step 10. Use the ABS function to find the absolute differences between the theoretical (Rank–1/*N*) and experimental CDF.

Model CDF from the left	Cummulative Normal Distribution Function (CDF)	difference from model (-)
Rank-1/N		
0	0,004339967	=ABS(K7-J7)
0,00507614	0,008478083	ABS(number)
0,01015228	0,009943615	
0,01522843	0,009943615	
0,02030457	0,011625636	
0,02538071	0,011625636	
0,03045685	0,011625636	
0,03553299	0,013549377	
0,04060914	0,015741882	
0,04568528	0,015741882	
0,05076142	0,018231964	
0,05583756	0,031800733	
0,06091371	0,031800733	
0,06598985	0,041230051	

→

Model CDF from the left	Cummulative Normal Distribution Function (CDF)	difference from model (-)
Rank-1/N		
0	0,004339967	0,00433997
0,00507614	0,008478083	0,00340194
0,01015228	0,009943615	0,00020867
0,01522843	0,009943615	0,00528481
0,02030457	0,011625636	0,00867893
0,02538071	0,011625636	0,01375507
0,03045685	0,011625636	0,01883122
0,03553299	0,013549377	0,02198362
0,04060914	0,015741882	0,02486725
0,04568528	0,015741882	0,0299434
0,05076142	0,018231964	0,03252946
0,05583756	0,031800733	0,02403683
0,06091371	0,031800733	0,02911297
0,06598985	0,041230051	0,0247598

Step 11. Use the MAX function to find the maximum difference ($D_{+/-}$ test statistic).

difference from model (+)	difference from model (-)	maximum difference (test statistic $D_{+/-}$)
0,00073617	0,00433997	=MAX(L9:M205)
0,0016742	0,00340194	
0,00528481	0,00020867	
0,01036095	0,00528481	
0,01375507	0,00867893	

Interpretation of the test results

Step 12. Finding the critical value.

In order to find the critical value (D_{crit}) for the desired percentage point (α), use the Massey's formulas (Massey, 1951) (Table 1 below). According to these formulas, 1.36 (α = 0.05) is divided by the square root of *N*, in our case *N* = 197.

Ranks	N	maximum difference (test statistic $D_{+/-}$)			Critical value for a = 0.05 $D_{0.05}$
0					
1	197	0,189809717			=Q18/SQRT(E9)
2	or				
3	197				
4	or				
5	197				

→

Critical value for a = 0.05 $D_{0.05}$
0,096895988

Table. The critical values for the one-sample KS test are presented.

Formula for critical values for different percentage points (α)					
α:	0,01	**0,05**	0,1	0,15	0,2
OVER 35	1,63	**1,36**	1,22	1,14	1,07
	____	____	____	____	____
	√ n	√ n	√ n	√ n	√ n

Compare the $D_{+/-}$ test statistic with D_{crit}. If $D_{+/-}$ is **lower** than D_{crit}, it means that the data are normally distributed. Otherwise, they are non-normally distributed. In our case, $D_{+/-}$ (0.1898) > D_{crit} (0.0968); therefore, example data are non-normally distributed.

Step 13. Calculate Kolmogorov's function.

To find the p-value, it is necessary to calculate the values of the Kolmogorov function (Kolmogoroff, 1933; Feller, 1948; Smirnov, 1948), where $z = D_{+/-} * \sqrt{n},\ z^2 = (D_{+/-})^2 * (\sqrt{n})^2 = (D_{+/-})^2 * n$, where *n* – sample size.

$$P = 1 - \mathrm{L(z)}, \text{ where}$$

$$\mathrm{L(z)} = 1 - 2 * \sum_{y=1}^{\infty} (-1)^{y-1} * e^{-2*y^2*z^2}$$

To calculate the values of Kolmogorov's function, follow the steps in Table 2 below from left to right.

Table. The calculation of the p-value for the KS test is presented.

1. Create *y* from 1 to 10	2. Type in Kolmogorov's formula: $(-1)^{y-1} * e^{-2*y^2*D_n^2*n}$	3. Propagate for all *y*

y
1
2
3
4
5
6
7
8
9
10

N	maximum difference (test statistic D+/-)	y	Kolmogorov's formula for p-value
197	0,189809717	1	=(-1)^(X9-1)*EXP(-2*(X9^2)*($N$9^2*$E$9))

y	Kolmogorov's formula for p-value
1	6,8426E-07
2	-2,1922E-25
3	3,2885E-56
4	-2,31E-99
5	7,596E-155
6	-1,17E-222
7	8,431E-303
8	0
9	0
10	0

Step 14. Finding the p-value.

To calculate the p-value using the formula:

$$P = 1 - \mathrm{L(z)}$$

sum Kolmogorov's function values for all *y*:

Kolmogorov's formula for p-value	p-value
	=1-(1-2*SUM(Y9:Y18))
6,84262E-07	
-2,19225E-25	
3,28853E-56	
-2,3097E-99	
7,5956E-155	
-1,1695E-222	
8,4313E-303	
0	
0	
0	

Compare the p-value to α (0.05). If *P* is greater than α, then the data are normally distributed. Otherwise, the data are not normally distributed. In our case, the p-value is less than 0.05. Therefore, we consider the data to be non-normally distributed, with a 0.00013685% probability of being wrong.

You can check the calculations using an online calculator ("Quest Graph™ Kolmogorov-Smirnov (K-S) Test Calculator." AAT Bioquest, Inc., 1 Dec. 2025, www.aatbio.com/tools/kolmogorov-smirnov-k-s-test-calculator)

P-value	P-value from online calculatior:
0,0000013685	0.0000013685

Supplementary S3

Calculation of the generalised Shapiro-Wilk normality test for lifespan data with partitioning into intervals. Step-by-step instruction.

Introductory notes

1. The generalized Shapiro-Wilk (SW) test is used to evaluate the probability that a given distribution is normal.
2. Survival data can be divided into intervals.
3. Two types of data can be used to divide into intervals:
   - lifespan data, where each number represents the age of the individual on the day of death (lifespan series format),
   - mortality data, where each number represents how many individuals died on a given day (deaths per day series format).

The SW test always interprets non-intervalized lifespan series as non-normally distributed due to fluctuations in daily mortality. For this and other reasons listed in the paper (reasons I-IV in the main text), we present the procedure for intervalized lifespan series with 12-18 intervals.
Step 1 involves preparing the survival data for the lifespan series (Sheet 1) and the deaths per day series (Sheet 2). The deaths per day series correspond to raw data. The lifespan series require conversion from deaths per day, unless your data are already in lifespan series format.
Step 2 involves determining the appropriate interval sizes for the lifespan series (Sheet 1) and the deaths per day series (Sheet 2). This step is different for the lifespan series and deaths per day series.
Step 3 involves partitioning the data into intervals to determine the frequency of phenotypes (i.e., the probability of death within a given time interval). This step is different for the lifespan series and deaths per day series.
Starting from step 4, the SW test statistics are calculated and the p-value is obtained in the same way for both lifespan series (Sheet 1) and deaths per day series (Sheet 2).

Method for lifespan series (step 1-3):
The first step is to convert the deaths per day series into a lifespan series. This conversion can be done using an algorithm:
1) If the deaths per day series has zero deaths on a given day, we do not add any number to the lifespan series.
2) If the deaths per day series has non-zero amount of deaths on a given day, then we add the number of the day to the lifespan series as many times as many deaths occurred on that day.

Step 1. Prepare data.

Paste the lifespan series, which is a row in which each number represents the age in days at which an individual from the sample died. Sort this row from lowest to highest.

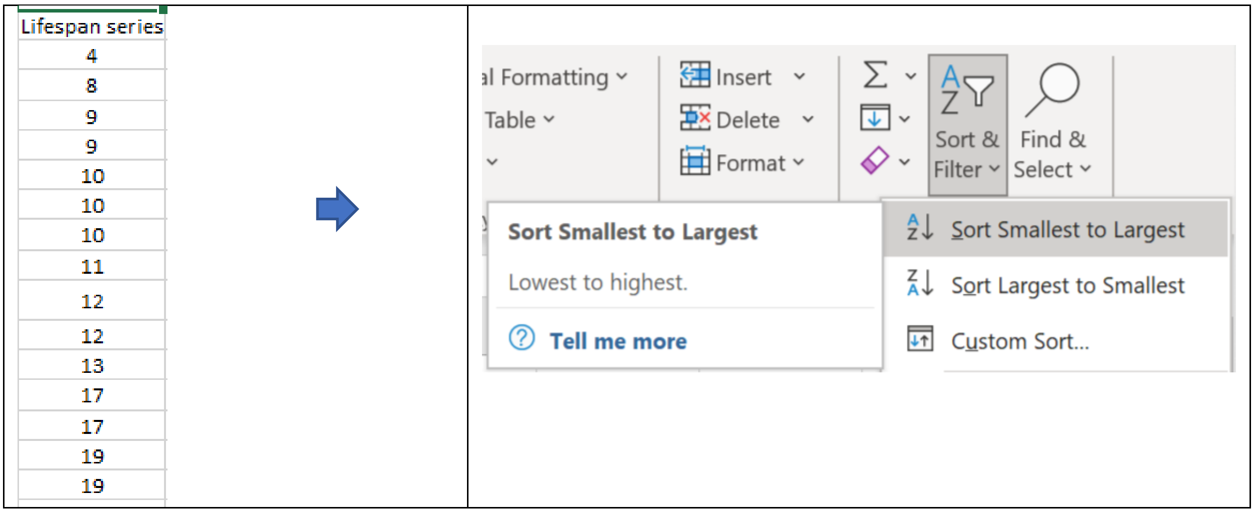

Step 2. Calculate convenient interval size.

Find the minimum and maximum ages of the lifespan series

**Min**		**Max**	
min age	=MIN(C17:C213)	min age	
max age	76	max age	=MAX(C17:C213)

Then, count the total number of flies that participated in the experiment.

N (total number of flies)	=COUNT(A4:A200)
	COUNT(value1; [value2]; ...)

Apply the Sturges formula to find the number of intervals.

min age	4		
max age	76		
N (total number of flies)	197		
**Sturges formula:**	$k = 1 + \log_2 N$	where k - is the number of intervals	
k	=1+LOG(H18;2)		

First, subtract the minimum lifespan from the maximum. Then, divide the difference by the number of intervals to find the size of each interval according to Sturges.

min age	4		
max age	76		
N (total number of flies)	197		
**Sturges formula:**	$k = 1 + \log_2 N$	where k - is the number of intervals	
k	8,622051819		
interval size:	=(H17-H16)/H21		

Take the most convenient interval size according to Sturges. For example, if you get intervals ranging from 4 to 8, take 5. If you get intervals ranging from 9 to 14, take 10, and so on. Repeat this procedure for each dataset. Select the same interval size for each sample.

Step 3. Partition the data into intervals.

1) Prepare the table for intervals:

If the first individual dies after the fourth day, skip the first interval (from 0 to 4) and start data partitioning from the second interval. Each subsequent interval begins on the last day of the previous interval plus one and ends on the first day of the interval plus four (see the figure below).

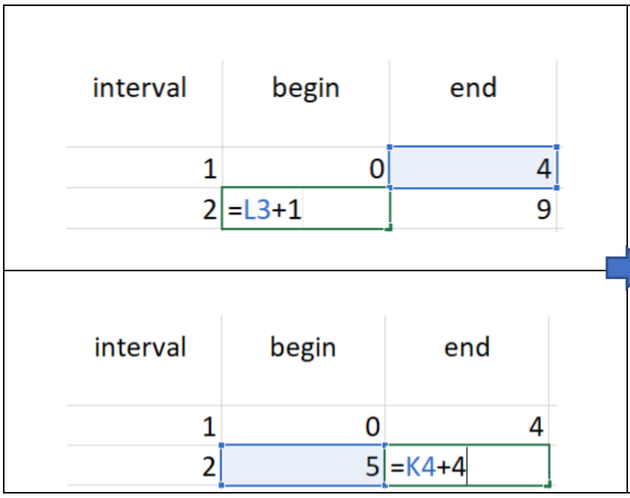

interval	begin	end
1	0	4
2	5	9
3	10	14
4	15	19
5	20	24

2) Find the number of individuals that fall into each interval (phenotype frequencies): Write the FREQUENCY function and use the sorted lifespan series from Step 1 as the first argument and the interval end values from the "End" column as the second argument.

Comment: The newest version of Excel will automatically propagate the row. If you are using an older version, select the cells corresponding to each interval from top to bottom. Press F2 to select the first cell, then press Shift+Ctrl+Enter to fill the selected cells with the corresponding frequency row.

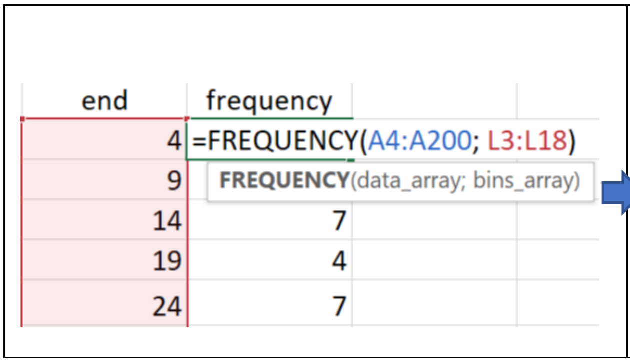

interval	begin	end	frequency
1	0	4	1
2	5	9	3
3	10	14	7
4	15	19	4
5	20	24	7

3) Calculate the percentage of individuals falling into each interval.

Comment: The test statistic can then be calculated using the number of individuals in each interval (see the "Phenotype Frequencies" column). For ease of understanding, however, the data can be converted to percentages. This conversion does not affect the calculation of the test statistic because it depends on the ratios between the number of individuals in each interval rather than the total number of individuals. Therefore, conversion to percentages is optional; if you prefer, the data can be presented as is.

frequency	probability of death per interval
1	0,51
3	=M5/SUM($M$4:$M$19) * 100
7	SUM(number1; [numb
4	2,03
7	3,55
16	8,12
4	2,03
17	8,63
6	3,05
7	3,55
51	25,89
20	10,15
19	9,64
21	10,66
13	6,60
1	0,51

interval	begin	end	frequency	probability of death per interval in %
1	0	4	1	0,51
2	5	9	3	1,52
3	10	14	7	3,55
4	15	19	4	2,03
5	20	24	7	3,55
6	25	29	16	8,12
7	30	34	4	2,03
8	35	39	17	8,63
9	40	44	6	3,05
10	45	49	7	3,55
11	50	54	51	25,89
12	55	59	20	10,15
13	60	64	19	9,64
14	65	69	21	10,66
15	70	74	13	6,60
16	75	79	1	0,51

Method for deaths per day series (step 1-3):
If the original data were a lifespan series from a patient study, for example, then it would be necessary to convert it into a series of deaths per day.
A deaths per day series is created. It goes from day 1 to day *n*. *n* is the maximum value in the lifespan series being converted. Each number in the lifespan series corresponds to a day of death in the death-per-day series. Therefore, if a given number occurs multiple times in the lifespan series, then that many deaths occurred on that day. The remaining days are filled with zeros. This algorithm can be performed using the FREQUENCY function in Excel (see Step 2 above under "Method" for the lifespan series).

Step 1. Prepare data.

The deaths per day series should be enumerated and not sorted. Each number in the series represents the number of deaths that occurred during the day.

Days	Deaths per day
1	0
2	0
3	0
4	1
5	0
6	0

Step 2. Calculate the convenient interval size.

Determine the minimum and maximum lifespans. The minimum lifespan is the first day on which mortality appeared.

Days	Deaths per day
1	0
2	0
3	0
4	1

min age	=A20
max age	76
N (total number of flies)	197

The maximum lifespan is the day on which the last individual died.

Days	Deaths per day
1	0
2	0
74	1
75	0
76	1

min age	4
max age	=A92
N (total number of flies)	197

*We hide cells three through seventy-three.

Use the SUM function (instead of the COUNT function) to calculate the total number of individuals in the lifespan series. This will give you the sample size needed to calculate the interval size.

Days	Deaths per day
1	0
2	0
74	1
75	0
76	1

min age	4
max age	76
N (total number of flies)	=SUM(B17:B92)

Use the Sturges formula to determine the correct number of intervals.

min age	4	
max age	76	
N (total number of flies)	197	
**Sturges formula:**	k = 1 + log2N,	where k – is the number of intervals
k	=1+LOG(F18;2)	

First, subtract the minimum lifespan from the maximum. Then, divide the difference by the number of intervals to find the size of each interval according to Sturges.

min age	4	
max age	76	
N (total number of flies)	197	
**Sturges formula:**	k = 1 + log2N	where k - is the number of intervals
k	8,622051819	
interval size:	=(H17-H16)/H21	

Take the most convenient interval size according to Sturges. For example, if you get intervals ranging from 4 to 8, take 5. If you get intervals ranging from 9 to 14, take 10, and

so on. Repeat this procedure for each dataset. Select the same interval size for each sample.

Step 3. Partition the data into intervals.

1) If the first individual dies after the fourth day, skip the first interval (from 0 to 4) and start data partitioning from the second interval. Each subsequent interval begins on the last day of the previous interval plus one and ends on the first day of the interval plus four (see the figure below).

interval	begin	end
1	0	4
2	=L3+1	9

interval	begin	end
1	0	4
2	5	=K4+4

interval	begin	end
1	0	4
2	5	9
3	10	14
4	15	19
5	20	24

2) Find the number of individuals that fall into each interval (phenotype frequencies): The number of individuals in each age interval should be calculated manually using the SUM function. Take into account deaths in each interval (0–4, 5–9, etc.). See the figure below. If done correctly, the result should be the same as with the lifespan series.

	A	B	J	K	L	M	N	O
3			interval	begin	end	frequency		
4	Sample	Day	1	0	4	=SUM(A5:A8)		
5	0	1	2	5	9	SUM(number1; [number2]; ...)		
6	0	2	3	10	14	7		
7	0	3	4	15	19	4		
8	1	4	5	20	24	7		
9	0	5	6	25	29	16		
10	0	6	7	30	34	4		
11	0	7	8	35	39	17		
12	1	8	9	40	44	6		
13	2	9	10	45	49	7		

	A	B	J	K	L	M	N	O
3			interval	begin	end	frequency		
4	Sample	Day	1	0	4	1		
5	0	1	2	5	9	=SUM(A9:A13)		
6	0	2	3	10	14	SUM(**number1**; [number2]; ...)		
7	0	3	4	15	19	4		
8	1	4	5	20	24	7		
9	0	5	6	25	29	16		
10	0	6	7	30	34	4		
11	0	7	8	35	39	17		
12	1	8	9	40	44	6		
13	2	9	10	45	49	7		

3) Calculate the percentage of individuals falling into each interval.
Comment: The test statistic can then be calculated using the number of individuals in each interval (see the "Phenotype Frequencies" column). For ease of understanding, however, the data can be converted to percentages. This conversion does not affect the calculation of the test statistic because it depends on the ratios between the number of individuals in each interval rather than the total number of individuals. Therefore, conversion to percentages is optional; if you prefer, the data can be presented as is.

frequency	probability of death per interval
1	0,51
3	=M5/SUM($M$4:$M$19) * 100
7	**SUM**(number1; [numb
4	2,03
7	3,55
16	8,12
4	2,03
17	8,63
6	3,05
7	3,55
51	25,89
20	10,15
19	9,64
21	10,66
13	6,60
1	0,51

interval	begin	end	frequency	probability of death per interval in %
1	0	4	1	0,51
2	5	9	3	1,52
3	10	14	7	3,55
4	15	19	4	2,03
5	20	24	7	3,55
6	25	29	16	8,12
7	30	34	4	2,03
8	35	39	17	8,63
9	40	44	6	3,05
10	45	49	7	3,55
11	50	54	51	25,89
12	55	59	20	10,15
13	60	64	19	9,64
14	65	69	21	10,66
15	70	74	13	6,60
16	75	79	1	0,51

Next, the same procedure is followed for both types of data (lifespan series and death per day series, starting from step 4 (calculating the denominator to obtain test statistics W).

Step 4. Find the denominator.

Copy the values of frequencies (frequency column) or probabilities (percentage column) and sort them from lowest to highest.

i (from 1 to n)	X (sample)
1	0,51
2	1,02
3	1,52
4	2,03
5	2,54
6	3,05
7	3,55
8	4,06
9	4,57
10	5,08
11	5,58
12	6,09
13	6,60
14	7,11
15	7,61
16	8,12

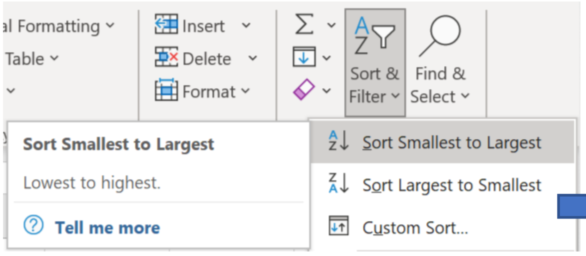

i (from 1 to n)	X (sample)
1	0,51
2	0,51
3	1,52
4	2,03
5	2,03
6	3,05
7	3,55
8	3,55
9	3,55
10	6,60
11	8,12
12	8,63
13	9,64
14	10,15
15	10,66
16	25,89

Find the deviation of each frequency from the mean (∑X/n, where X is the sorted sample).

i (from 1 to n)	X (sample)	xi-(∑X/n)
1	=Q4-SUM($Q$4:$Q$19)/$P$19	
2	0,51	-5,74
3	1,52	-4,73
4	2,03	-4,22
5	2,03	-4,22
6	3,05	-3,20
7	3,55	-2,70
8	3,55	-2,70
9	3,55	-2,70
10	6,60	0,35
11	8,12	1,87
12	8,63	2,38
13	9,64	3,39
14	10,15	3,90
15	10,66	4,41
16	25,89	19,64

i (from 1 to n)	X (sample)	xi-(∑X/n)
1	0,51	$P$19
2	0,51	-5,74
3	1,52	-4,73
4	2,03	-4,22
5	2,03	-4,22
6	3,05	-3,20
7	3,55	-2,70
8	3,55	-2,70
9	3,55	-2,70
10	6,60	0,35
11	8,12	1,87
12	8,63	2,38
13	9,64	3,39
14	10,15	3,90
15	10,66	4,41
16	25,89	19,64

i (from 1 to n)	X (sample)	xi-(∑X/n)
1	0,51	-5,74
2	0,51	-5,74
3	1,52	-4,73
4	2,03	-4,22
5	2,03	-4,22
6	3,05	-3,20
7	3,55	-2,70
8	3,55	-2,70
9	3,55	-2,70
10	6,60	0,35
11	8,12	1,87
12	8,63	2,38
13	9,64	3,39
14	10,15	3,90
15	10,66	4,41
16	25,89	19,64

Find the squares of the calculated values.

xi-(∑X/n)	(xi-(∑X/n))^2
-5,74	=R4^2
-5,74	
-4,73	
-4,22	
-4,22	
-3,20	
-2,70	
-2,70	
-2,70	
0,35	
1,87	
2,38	
3,39	
3,90	
4,41	
19,64	

i (from 1 to n)	X (sample)	xi-(∑X/n)	(xi-(∑X/n))^2
1	0,51	-5,74	32,97
2	0,51	-5,74	32,97
3	1,52	-4,73	22,35
4	2,03	-4,22	17,80
5	2,03	-4,22	17,80
6	3,05	-3,20	10,27
7	3,55	-2,70	7,27
8	3,55	-2,70	7,27
9	3,55	-2,70	7,27
10	6,60	0,35	0,12
11	8,12	1,87	3,50
12	8,63	2,38	5,66
13	9,64	3,39	11,52
14	10,15	3,90	15,23
15	10,66	4,41	19,45
16	25,89	19,64	385,66

The denominator will be the sum of all these squares.

(xi-(∑X/n))^2	SUM
32,97	=SUM(S4:S19)
32,97	
22,35	
17,80	
17,80	
10,27	
7,27	
7,27	
7,27	
0,12	
3,50	
5,66	
11,52	
15,23	
19,45	
385,66	

i (from 1 to n)	X (sorted sample)	xi-(∑X/n)	(xi-(∑X/n))^2	SUM
1	0,51	-5,74	32,97	597,14
2	0,51	-5,74	32,97	
3	1,52	-4,73	22,35	
4	2,03	-4,22	17,80	
5	2,03	-4,22	17,80	
6	3,05	-3,20	10,27	
7	3,55	-2,70	7,27	
8	3,55	-2,70	7,27	
9	3,55	-2,70	7,27	
10	6,60	0,35	0,12	
11	8,12	1,87	3,50	
12	8,63	2,38	5,66	
13	9,64	3,39	11,52	
14	10,15	3,90	15,23	
15	10,66	4,41	19,45	
16	25,89	19,64	385,66	

Step 5. Calculate the value of *m* (the median of a series of indices).

If the number of intervals (n) is even, then m is equal to $n/2$, if the number of intervals is odd, then $m=(n–1)/2$.

i (from 1 to n)	m	=(R31/2)
1		
2		
3		
4		
5		
6		
7		
8		
9		
10		
11		
12		
13		
14		
15		
16		

i (from 1 to n)		
1		
2		
3		
4		
5	m	8,00
6		
7		
8		
9		
10		
11		
12		
13		
14		
15		
16		

Step 6. Calculate the numerator.

Take the corresponding $a_{n–i+1}$ coefficient from Table 5 of the original article by Shapiro–Wilk (1965). For example, if the sample has 16 intervals, then the coefficients are taken from the 16th column of Table 5 and inserted into Excel.

Table 5 Shapiro–Wilk (1965)

i \ n	11	12	13	14	15	16
**1**	0·5601	0·5475	0·5359	0·5251	0·5150	0·5056
**2**	·3315	·3325	·3325	·3318	·3306	·3290
**3**	·2260	·2347	·2412	·2460	·2495	·2521
**4**	·1429	·1586	·1707	·1802	·1878	·1939
**5**	·0695	·0922	·1099	·1240	·1353	·1447
**6**	0·0000	0·0303	0·0539	0·0727	0·0880	0·1005
**7**	—	—	·0000	·0240	·0433	·0593
**8**	—	—	—	—	·0000	·0196
**9**	—	—	—	—	—	—
**10**	—	—	—	—	—	—

m	8
i (from 1 to m)	a_{n-i+1}
1	0,5056
2	0,329
3	0,2521
4	0,1939
5	0,1447
6	0,1005
7	0,0593
8	0,0196

Return to the sorted sample series from Step 4 and calculate the difference between the first and last sorted probabilities. In this case, interval #8 is the median interval, so no operation is required for this interval. The overall procedure can be expressed by the following formula: $(x_{n–i+1} – x_i)$

i (from 1 to n)	X (sample)	
1	0,51	
2	0,51	
3	1,52	
4	2,03	
5	2,03	
6	3,05	$(x_{n-i+1} - x_i)$
7	3,55	=Q19-Q4
8	3,55	
9	3,55	
10	6,60	
11	8,12	
12	8,63	
13	9,64	
14	10,15	
15	10,66	
16	25,89	

i (from 1 to n)	X (sample)	
1	0,51	
2	0,51	
3	1,52	
4	2,03	
5	2,03	
6	3,05	$(x_{n-i+1} - x_i)$
7	3,55	25,38
8	3,55	=Q18-Q5
9	3,55	
10	6,60	
11	8,12	
12	8,63	
13	9,64	
14	10,15	
15	10,66	
16	25,89	

m	8	
i (from 1 to m)	a_{n-i+1}	$(x_{n-i+1} - x_i)$
1	0,5056	25,38
2	0,329	10,15
3	0,2521	8,63
4	0,1939	7,61
5	0,1447	6,60
6	0,1005	5,08
7	0,0593	3,05
8	0,0196	3,05

Multiply these values by the corresponding a_{n-i+1} coefficients (see the beginning of the step 6).

i (from 1 to m)	a_{n-i+1}	$(x_{n-i+1} - x_i)$	$a_{n-i+1}*(x_{n-i+1} - x_i)$
1	0,51	25,38	=W10*X10
2	0,33	10,15	
3	0,25	8,63	
4	0,19	7,61	
5	0,14	6,60	
6	0,10	5,08	
7	0,06	3,05	
8	0,02	3,05	

m	8		
i (from 1 to m)	a_{n-i+1}	$(x_{n-i+1} - x_i)$	$a_{n-i+1}*(x_{n-i+1} - x_i)$
1	0,5056	25,38	12,83
2	0,329	10,15	3,34
3	0,2521	8,63	2,18
4	0,1939	7,61	1,48
5	0,1447	6,60	0,95
6	0,1005	5,08	0,51
7	0,0593	3,05	0,18
8	0,0196	3,05	0,06

Find the sum of the obtained values.

$a_{n-i+1}*(x_{n-i+1} - x_i)$	SUM
=SUM(Y10:Y17)	
2,18	
1,48	
0,95	
0,51	
0,18	
0,06	

$a_{n-i+1}*(x_{n-i+1} - x_i)$	SUM
12,83	21,53
3,34	
2,18	
1,48	
0,95	
0,51	
0,18	
0,06	

To obtain the numerator, calculate the square of this sum.

SUM	SQUARE
21,53	=Z10^2

Numerator					
m	8,00				
i (from 1 to m)	a_{n-i+1}	$(x_{n-i+1} - x_i)$	$a_{n-i+1}*(x_{n-i+1} - x_i)$	SUM	SQUARE
1,00	0,51	25,38	12,83	21,53	463,53
2,00	0,33	10,15	3,34		
3,00	0,25	8,63	2,18		
4,00	0,19	7,61	1,48		
5,00	0,14	6,60	0,95		
6,00	0,10	5,08	0,51		
7,00	0,06	3,05	0,18		
8,00	0,02	3,05	0,06		

Step 7. Calculate the test statistics (*W*).

To obtain W, divide the numerator by the denominator.

Denominator			
SUM		W	=AA10/T4
597,14			
	Numerator		
	SQUARE		
	463,53		

W	0,776

Interpretation of the results

Interpretation via percentage points. Use the W values corresponding to the percentage point values in Table 6 from Shapiro–Wilk (1965). The probability of rejecting the null hypothesis that our distribution is normal will be evaluated by comparing our W value to the W values in Table 6. For example, our W value is 0.776. The corresponding W values for the percentage points (0.01, 0.02, 0.05, 0.1, 0.5, 0.9, 0.95, 0.98, and 0.99) are 0.844, 0.863, 0.887, 0.906, 0.952, 0.976, 0.981, 0.985, and 0.987, respectively. 0.776 is less than any of these values. Therefore, the given distribution is not normal, with a less than 1% (<0.01) chance of being normal.

Interpretation by p-value. Royston (1993) normalization transformations can be used to calculate the p-value. See the "Materials and Methods" section for details. The calculated p-value, or the probability that the distribution is normal, is 0.0013 according to the Royston formula.

p-value interpretation	
μ_z	-3,008749
σz	0,5034692
z	3,0021728
p-value	0,0013403

You can check the calculations using an online calculator (www.statskingdom.com/shapiro-wilk-test-calculator.html). For instance, Statistic Kingdom provides a W value of 0.772 and a p-value of 0.001183 for the same sample.

Parameter	Value
P-value	0.001183
W	0.772

Supplementary S4

Python script instruction

The script contains code that perform the following operations:

1. Represent the survival curves created using the Kaplan-Meier method.
2. Convert the survival data (mortality series, deaths per day) to a lifespan series using the ‘convert_deaths_per_day_to_lifespan’ function.
3. Combine the adjacent days into groups of five days to create a frequency/probability series of phenotypes by lifespan (‘sample_to_Intervals’ function is being used for that). The series allows for easier comparison of the two samples (control and mutant) on the bar chart.
4. Represent the frequency series of phenotypes by lifespan using a bar chart.
5. Divide the lifespan series (the control and the mutant) into intervals using Sturges' rule (for an accurate comparison, it is essential that the samples have the same number of intervals, although the sizes of the intervals may vary).
6. Calculate the Shapiro-Wilk test statistics (W) and probability (*P*) using the 1965 Shapiro-Wilk method and the 1993 Royston method, respectively, for intervalized frequency series.
7. Using the initial lifespan, calculate the normal distribution and superimpose it on the frequency series/distributions for the mutant and control groups. This allows for a quick, formal comparison of the mutant and control group distributions.
8. Using the initial lifespan, calculate the β-distribution, then superimpose it on the frequency series/distributions for the mutant and control groups. The applied β-distribution allows for a formal, quick comparison of the frequency series/distributions of the mutant and control groups.
9. Calculate the normal distribution for the initial lifespan series, as well as for the ideal normal lifespan series (data on the initial mortality day, final mortality day, and experimental sample size are used to construct it) and superimpose these two normal distributions on the experimental frequency series (5-day intervals) for the control and for the mutant groups separately.
10. If the β-distribution selected in automatic mode does not adequately describe the frequency series, select the β-distribution graph manually. The code will generate multiple

β-distribution curves for each experimental sample using all possible options for calculating p and q. It will also use the intervalized and original lifespan series, as well as the mode or median of the series. Different values of 'Initial_day' (day of the interval from 0 to 4) will also be used. To manually select the β-distribution shape, use the settings in the ADVANCED SETTINGS section and select the parameters that best describe the frequency series.

11. Calculate the Shapiro-Wilk test statistics (W) and probability (*P*) using the 1965 Shapiro-Wilk method and the 1993 Royston method, respectively, for each sample intervalized by five days.

12. Calculate the SW and KS tests on the 5-day interval series and on the non-interval (original) series. Generate a table displaying the W or D_n and p-value for each test.

DATA INPUT

There are two ways in which you can input the deaths-per-day data: either you can upload the *.xml file or enter the data manually according to the example.

Input via file

If you use a file to enter the data, the contents of the first cell will resemble the example shown in Figure below.

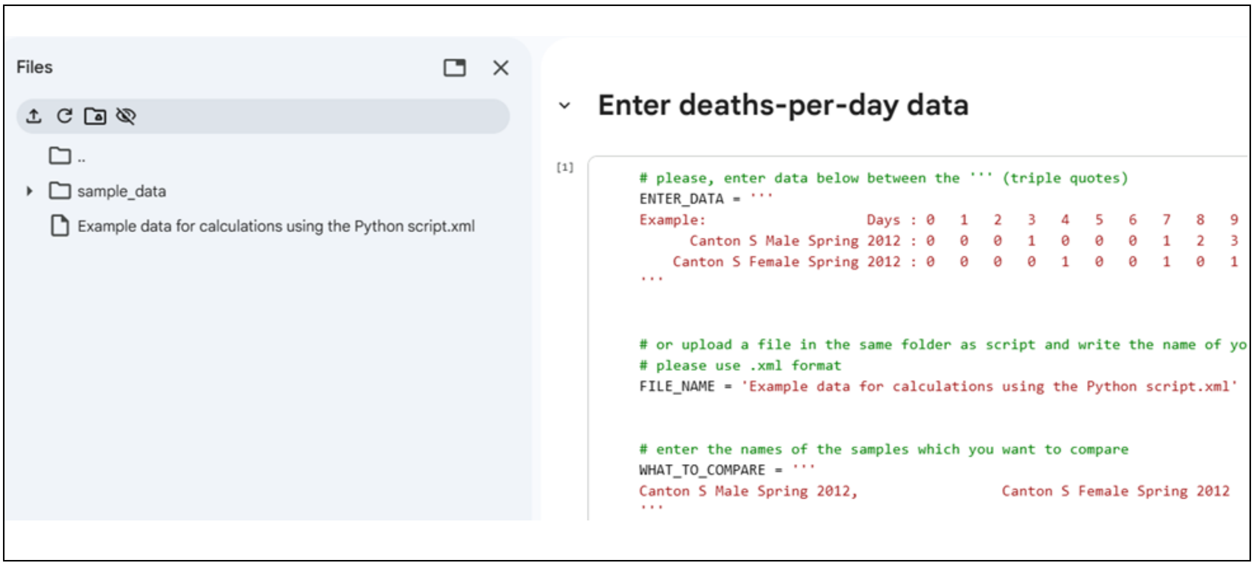

Figure. An example of the data entry cell using a file.

- The 'ENTER_DATA' variable can contain data or be empty; the 'ENTER_DATA' variable is prioritized over the 'FILE_NAME' variable.
- The 'FILE_NAME' variable refers to an existing file.
- The WHAT_TO_COMPARE variable contains the cohorts which are present in the file.

1) Create a new Excel document. Enter the deaths-per-day data as shown in Figure below.

	A	B	C	D	E	BW	BX	BY	CI	CJ	CK
1	Days	0	1	2	3	73	74	75			
2	Canton S Male Spring 2012	0	0	0	1	1	0	1			
3	Canton S Female Spring 2012	0	0	0	0	5	2	11	0	0	1
4											

Example genotype

Figure. *.xml file with experimental data opened in Excel.

Comments: Column A must contain the names of the test groups. Rows containing 'day' will not be analyzed (as specified in the script algorithm), therefore the genotype name of the sample must not contain 'day'. When specifying the genotype, do not use the character ‘$’. Starting from column B, enter the numerical mortality values day by day, beginning on day zero, in the corresponding row of the genotype. Cells containing letters or symbols instead of numbers, or cells containing a combination of numbers and letters, will not be analyzed. When a cell containing a letter or symbol is encountered, the result will be shifted. For example, if a letter is encountered on day 3, the cell corresponding to day 3 will be ignored, and the cell corresponding to day 4 will be interpreted as day 3. Similarly, if an empty cell is found on day 3, the result will be shifted by one day. However, if an empty cell is found at the start or end of the row and the rest of the row is intact, no shift will occur.

2) Name the sheet to signify the genotype or comparison group being studied (an experiment). It is best to place data from different experiments on different sheets, as the script will only compare pairs from the same sheet (see next section 3, ‘STEPS TO TAKE AFTER THE DATA INPUT’).

3) Save the file in *.xml format (Table .XML 2003).

4) Upload the script file to your Google Drive and open the data in it as follows:

4.1 Open Google Drive.

4.2 Move the script file into a folder.

4.3 Open the script file as follows: right-click on the file and select ‘Open with,’ then ‘Google Colaboratory’.

4.4 In the ‘**Enter death-per-day data**’ cell, enter the name of the *.xml file containing your data (Figure below).

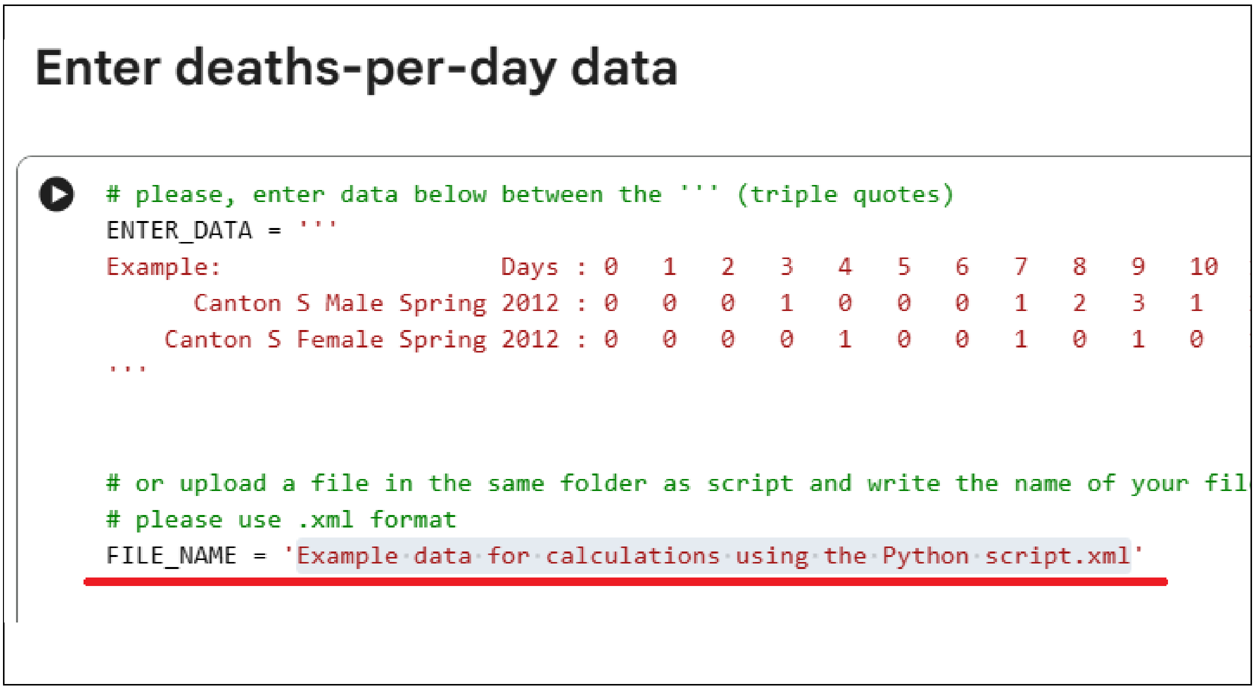

Figure. Entering the name of the *.xml file.

Click on the ‘Files’ tab (folder icon). A menu will open. To upload a file with your data, click on the image of a sheet with an upward arrow (Figure below).

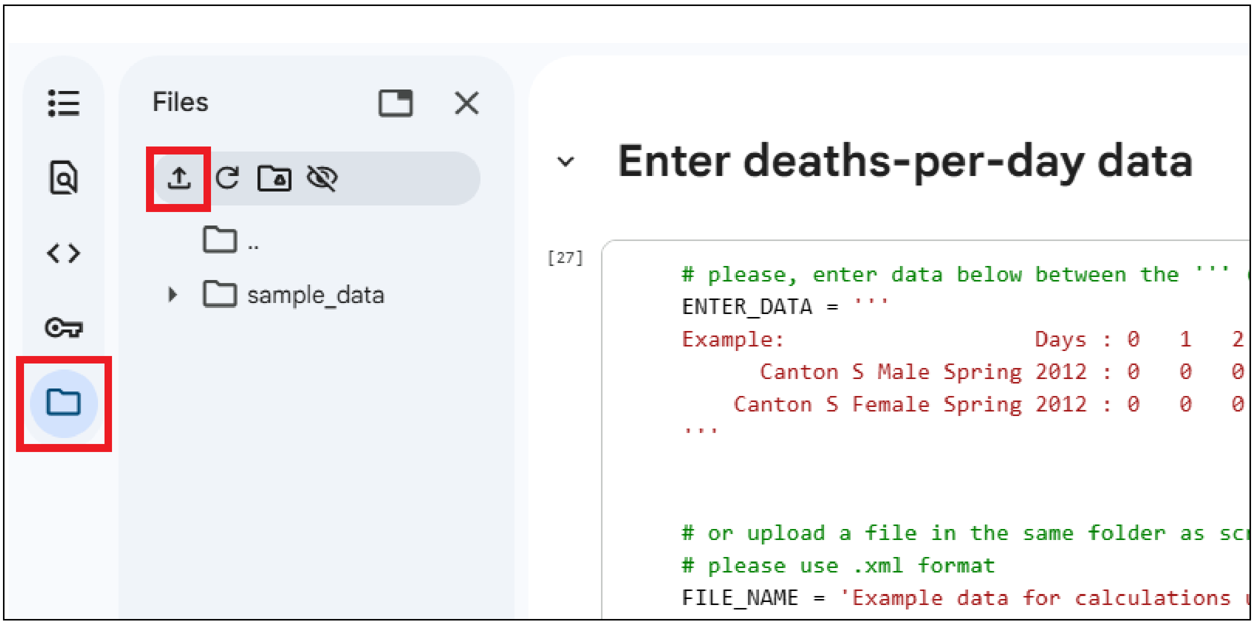

Figure. Uploading the file into Colab Enviroment.

Manual input

If you enter the data manually, the cell will resemble the example shown in Figure below.

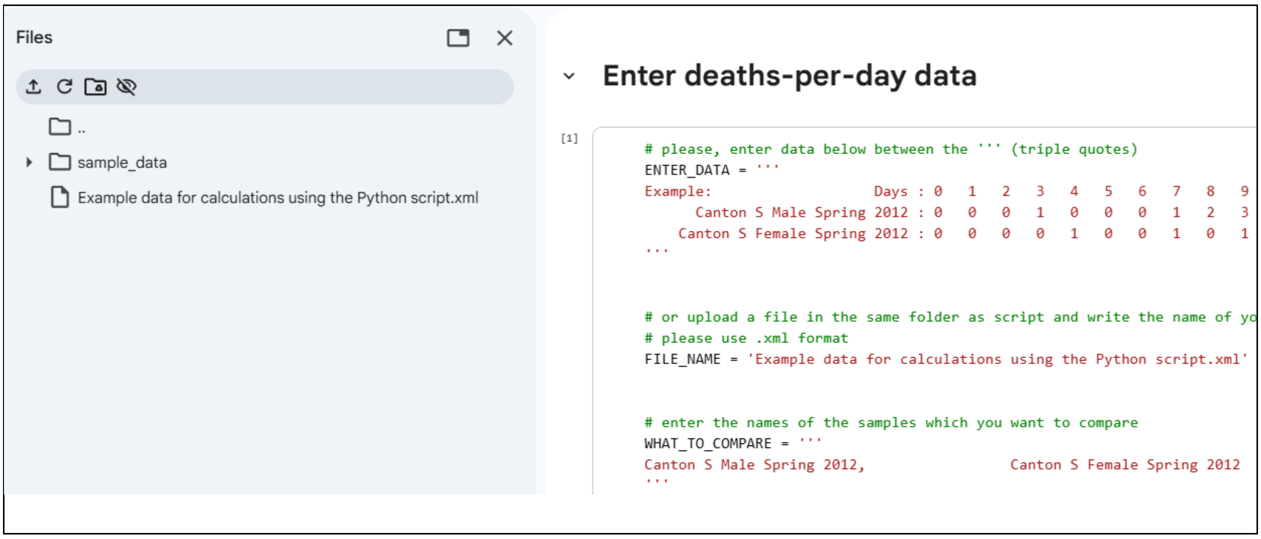

Figure. An example of the data entry cell using a manual input.

- The 'ENTER_DATA' variable contains data.
- The FILE_NAME variable is either empty or refers to a non-existent file.
- The 'WHAT_TO_COMPARE' variable contains the cohorts which are present in 'ENTER_DATA'.

If you are using manual input, make sure that the FILE_NAME variable does not refer to an existing file (Figure below).

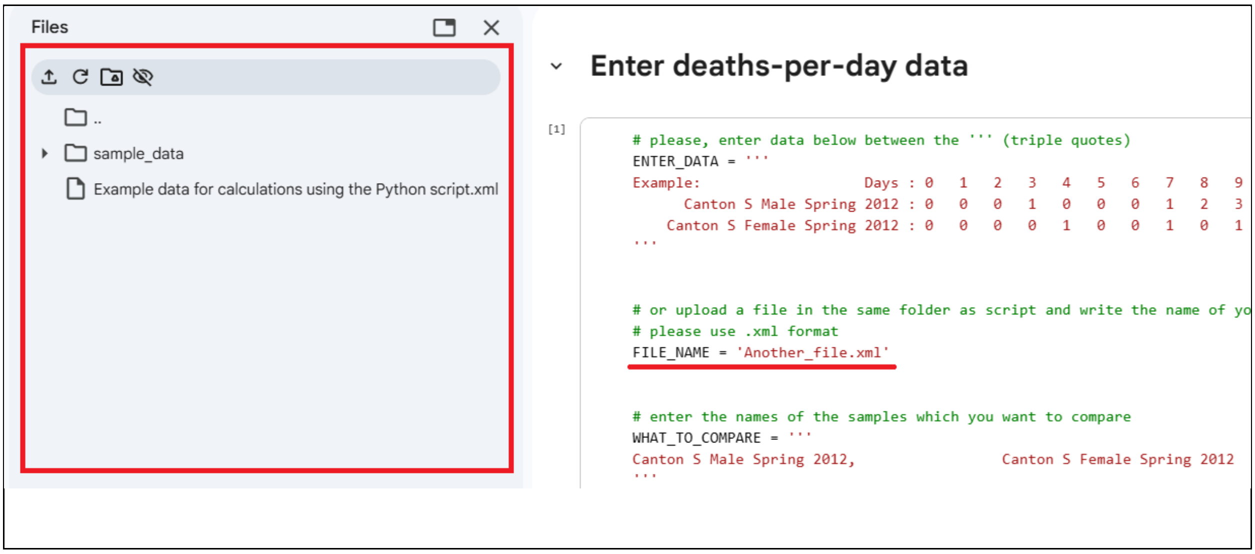

Figure. The figure shows manual input. The variable 'FILE_NAME' contains the file name. However, since the specified file was not uploaded, manual input is performed instead. Data is read from the 'ENTER_DATA' variable.

To enter data manually, edit the 'ENTER_DATA' variable in the **'Enter death-per-day data'** cell by entering the data according to the example (Figure below).

Enter deaths-per-day data

```
# please, enter data below between the ''' (triple quotes)
ENTER_DATA = '''
Example:                    Days : 0   1   2   3   4   5   6   7   8   9   10  11  12  13  14  15
         Canton S Male Spring 2012 : 0   0   0   1   0   0   0   1   2   3   1   2   1   0   0   0
      Canton S Female Spring 2012 : 0   0   0   0   1   0   0   1   0   1   0   1   0   0   3   0
'''
```

Figure. Manual input of deaths-per-day data for example genotype.

Data can be pasted from an Excel file as follows:
1. Copy a series of values from an Excel file into a *.txt file or the address bar of your browser.
2. Copy the data from the *.txt file or address bar and then paste it into the code.

3. STEPS TO TAKE AFTER THE DATA INPUT

Edit the 'WHAT_TO_COMPARE' variable to specify which cohorts should be paired for comparison. This is necessary for both input methods, whether you enter the data manually or use a file. Cohorts are compared in pairs; each cohort specified in 'WHAT_TO_COMPARE' variable should be separated by a comma. Each pair of cohorts should be entered on a new line.

WHAT_TO_COMPARE = ‘’’
CONTROL SAMPLE_1 NAME, MUTANT SAMPLE_1 NAME
CONTROL SAMPLE_2 NAME, MUTANT SAMPLE_2 NAME
CONTROL SAMPLE_3 NAME, MUTANT SAMPLE_3 NAME...
‘’’

An example is shown in the figure below.

Enter deaths-per-day data

```
# please, enter data below between the ''' (triple quotes)
ENTER_DATA = '''
Example:                   Days : 0   1   2   3   4   5   6   7   8   9   10  11  12  13  14  15
      Canton S Male Spring 2012 : 0   0   0   1   0   0   0   1   2   3   1   2   1   0   0   0
    Canton S Female Spring 2012 : 0   0   0   0   1   0   0   1   0   1   0   1   0   0   3   0
'''


# or upload a file in the same folder as script and write the name of your file
# please use .xml format
FILE_NAME = 'Example data for calculations using the Python script.xml'


# enter the names of the samples which you want to compare
WHAT_TO_COMPARE = '''
Canton S Male Spring 2012,                Canton S Female Spring 2012
'''

# FOR CUSTOM GRAPHS PLEASE CHECK THE "ADVANCED SETTINGS" CELL
```

Figure. One pair of cohorts for comparison (Canton S Male Spring 2012 and Canton S Female Spring 2012).

Next, click 'Run All' (Figure below) or press Ctrl+F9, and wait a moment.

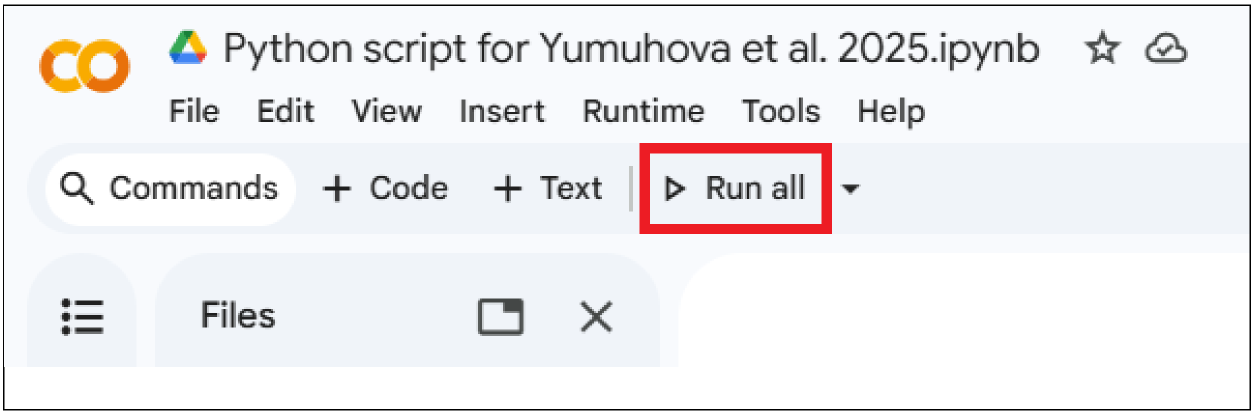

The resulting tables will appear in the 'Files' tab and the graphs in the corresponding cells. The output consists of three *.txt files and seven *.png image files for each comparison pair. In addition, there are five graphs for the β-distribution shape selection for each pair. To save the graphs, right-click and select 'Save as'.

OUTPUT of the script

***.txt files:**

1. The file 'Calculation of SW and KS tests on 5-day intervalized and non-intervalized (original) lifespan series.txt' contains a comparison of the results from the Shapiro-Wilk and Kolmogorov-Smirnov tests for non-intervalized (original) and intervalized (5-day) data (four variants of calculation).

2. The file ‘Shapiro-Wilk test, performed on lifespan data divided into 5-day intervals.txt’ contains the results of the Shapiro-Wilk test calculated on data intervalized by 5 days.

3. The file ‘Shapiro-Wilk test, performed on the lifespan data divided into intervals according to Sturges's rule.txt’ contains data that has been divided into intervals according to Sturges's rule and has been calculated using the Shapiro-Wilk test.

The figure below shows a demonstration of the output of *.txt files.

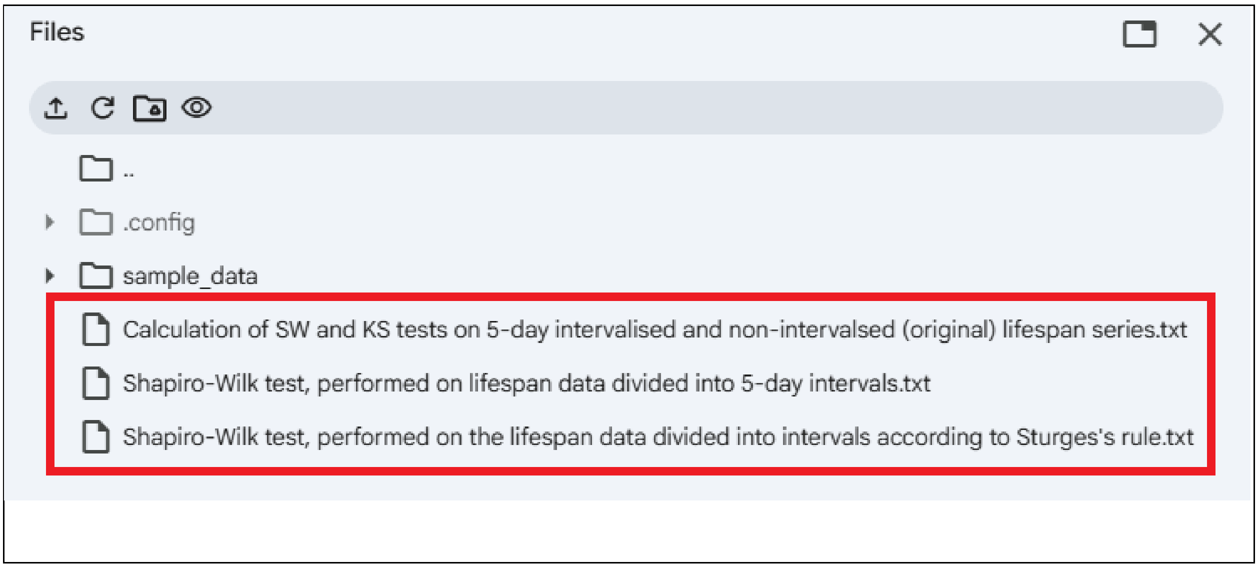

Figure. The results of executing the script are presented as *.txt files.

Files in *.txt format have to be downloaded to your computer and opened in Excel. To do this, right-click on the file and select ‘Download.’ Then, go to the folder where you saved the file, right-click on it, select ‘Open with,’ and choose Excel.

The file ‘Calculation of SW and KS tests on 5-day intervalized and non-intervalized (original) lifespan series.txt’ contains a table with the following columns:

A. Names of genotypes and cohorts.
B. Statistics of the SW test calculated on non-intervalized data.
C. p-value of the SW test calculated on non-intervalized data.
D. Statistics of the SW test calculated on data intervalized by five days.
E. p-value of the SW test calculated on data intervalized by five days.
F. Statistics of the KS test calculated on non-intervalized data.
G. p-value of the KS test calculated on non-intervalized data.
H. Statistics of the KS test calculated on data intervalized by five days.
I. p-value of the KS test calculated on data intervalized by five days.

	A	B	C	D	E	F	G	H	I	J
1	Calculation of SW and KS tests on 5-day intervalised and non-intervalsed (original) lifespan series									
2		SW-test				KS-test				
3		non-intervalized		intervalized		non-intervalized		intervalized		
4		W	p-value	W	p-value	Dn	p-value	Dn	p-value	
5	Genotype									
6	Canton S Male Spring 2012	0.93	3.21E-08	0.72	0.00027	0.19	1.06E-06	0.22	0.43832	
7	Canton S Female Spring 2012	0.92	6.89E-08	0.86	0.01087	0.11	0.04031	0.25	0.20994	
8										
9										

Calculation of SW and KS tests

The file ‘Shapiro-Wilk test, performed on lifespan data divided into 5-day intervals.txt’ contains a table with the following columns:

A. Names of genotypes and cohorts.
B. Sample sizes.
C. Maximum lifespan.
D. SW test statistics.
E. p-value.
F. Interpretation of SW test results.
G. Interval width.
H. Number of intervals.
J. Starting with column J – intervalized data (number of individuals that died in a given 5-day interval, where 0..4, 5..9, etc. are the interval boundaries).

	A	B	C	D	E	F	G	H	I	J	K	L
1	Shapiro-Wilk test, performed on lifespan data divided into 5-day intervals											
2	Sample name	Number of flies	Maximum lifespan	SW test statistics	p-value	Conclusion	Width of the intervals	Number of intervals		Intervalised data		
3										0..4	5..9	10..14
4	Genotype											
5	Canton S Male Spring 2012	197	75	0.72	<0.001	not norm with alpha = 0.05	5	16		1	6	4
6	Canton S Female Spring 2012	163	87	0.86	0.011	not norm with alpha = 0.05	5	18		1	2	4
7												
8												
9												

Shapiro-Wilk test, performed on

The file ‘Shapiro-Wilk test, performed on the lifespan data divided into intervals according to Sturges's rule.txt’ contains a table with the following columns:

A. Names of genotypes and cohorts.
B. Sample sizes.
C. Maximum lifespan.
D. SW test statistics.
E. p-value.
F. Interpretation of SW test results.
G. Number of intervals calculated using Sturges' rule for each sample (initial).
H. Width of intervals calculated according to Sturges' rule for each sample (initial).
I. Number of intervals after calculating the minimum number of intervals for a pair (recalculated number).

J. The width of the intervals obtained after calculating the minimum number of intervals for a pair (recalculated width).
L. Starting with column L – intervalized data (data on the number of individuals that died in a given interval. The interval numbering is given above the data).

	A	B	C	D	E	F	G	H	I	J	K	L	M	N
1	Shapiro-Wilk test, performed on the lifespan data divided into intervals according to Sturges's rule													
2	Sample name	Number of flies	Maximum lifespan	SW test statistics	p-value	Conclusion	Sturges's rule number of intervals	Sturges's rule width of the intervals	Number of intervals equalized between samples	Final width of the intervals		Intervalised data		
3												1	2	3
4	Genotype													
5	Canton S Male Spring 2012	197	75	0.91	0.368	norm with alpha = 0.05	8.62	8.81	9	9.1		4	9	17
6	Canton S Female Spring 2012	163	87	0.86	0.104	norm with alpha = 0.05	8.35	10.54	9	10.54		3	7	6
7														
8														
9														

Shapiro-Wilk test, performed on

***.png files:**

The *.png files in the cell output contain images that show comparisons of genotypes marked in the first cell (‘WHAT_TO_COMPARE’). The number of files corresponds to the number of comparison pairs. To view the *.png files, go to the desired graph type (Figure below) and expand it by clicking the arrow in the corner of the cell (the figure after figure below).

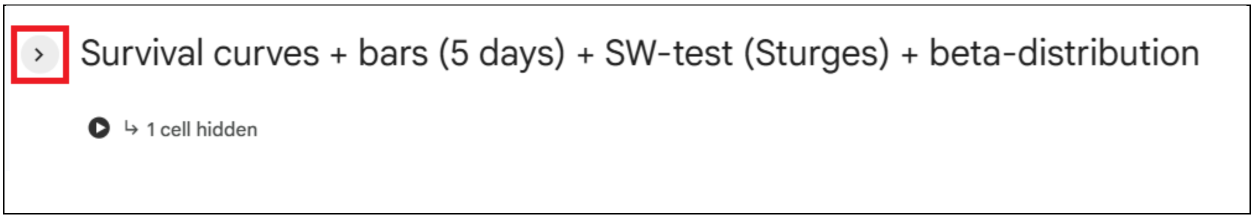

Figure. Demonstrates how to open a cell to view the output.

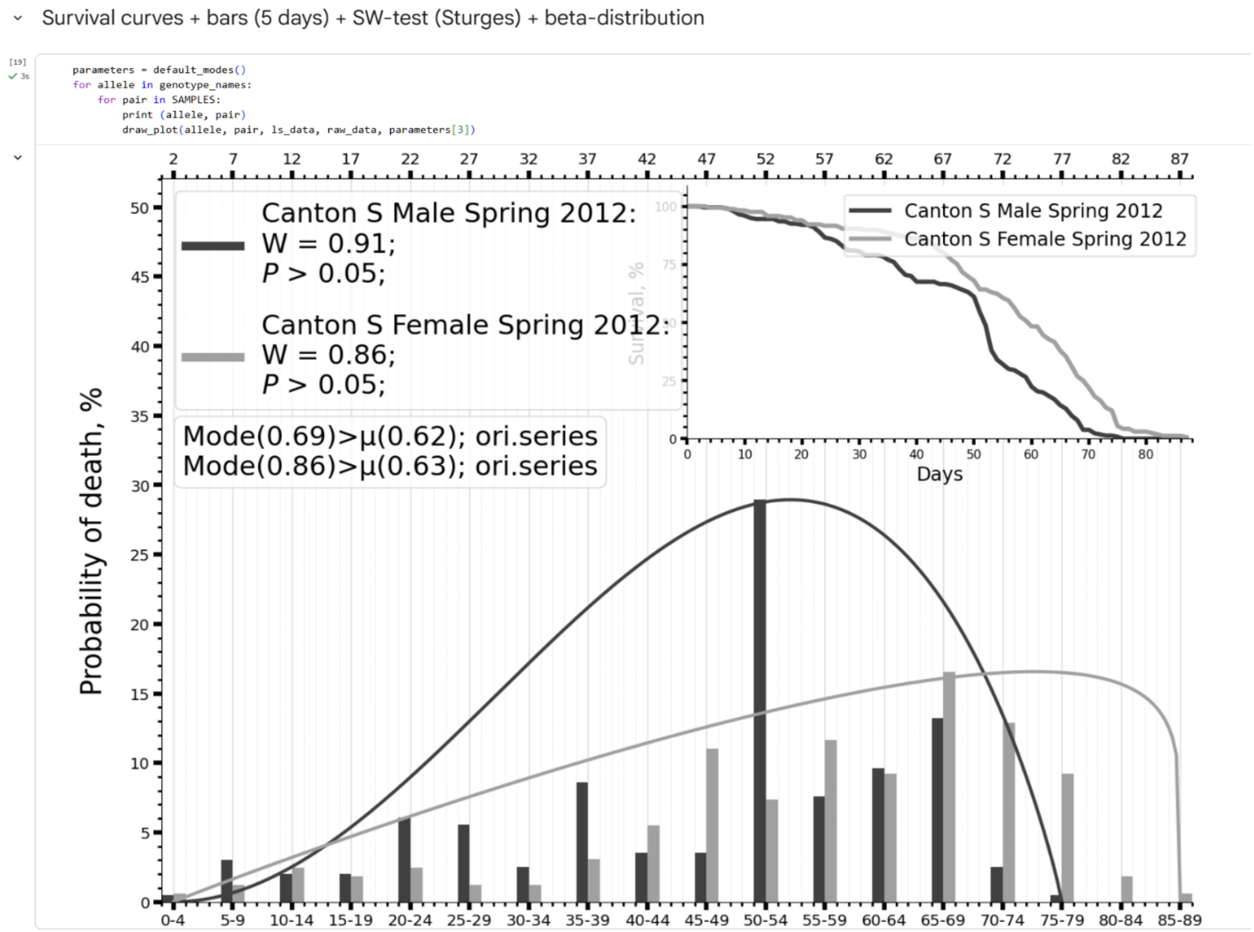

Figure. The example of the output is presented, showing the pair of frequency/probability series of phenotypes by lifespan, which were described using a β-distribution. In the upper left corner, the SW test is calculated for a pair of samples using Sturges' rule (equal number of intervals between the compared samples when they have different lengths).

A total of seven basic graphs will be plotted for each pair of samples:

1. Survival curves.
2. Survival curves with bars (5-day intervals).
3. Survival curves + bars (5-day intervals) + SW-test (Sturges' intervalization).
4. Survival curves + bars (5-day intervals) + SW-test (Sturges' intervalization) + normal distributions for the control and mutant groups.
5. Survival curves + bars (5-day intervals) + SW test (Sturges' intervalization) + β-distributions (by decision tree) for the control and mutant groups.
6. Survival curves + bars + SW-test (Sturges' intervalization) + normal and ideal normal distributions for the control group.
7. Survival curves + bars + SW-test (Sturges' intervalization) + normal and ideal normal distributions for the mutant group.

Also, five additional graphs will be plotted for the manual selection of β-distribution curves for each pair of samples (one graph for each 'Initial_day' value, i.e., the day of the interval from 0 to 4). All graphs are located in the corresponding cells.

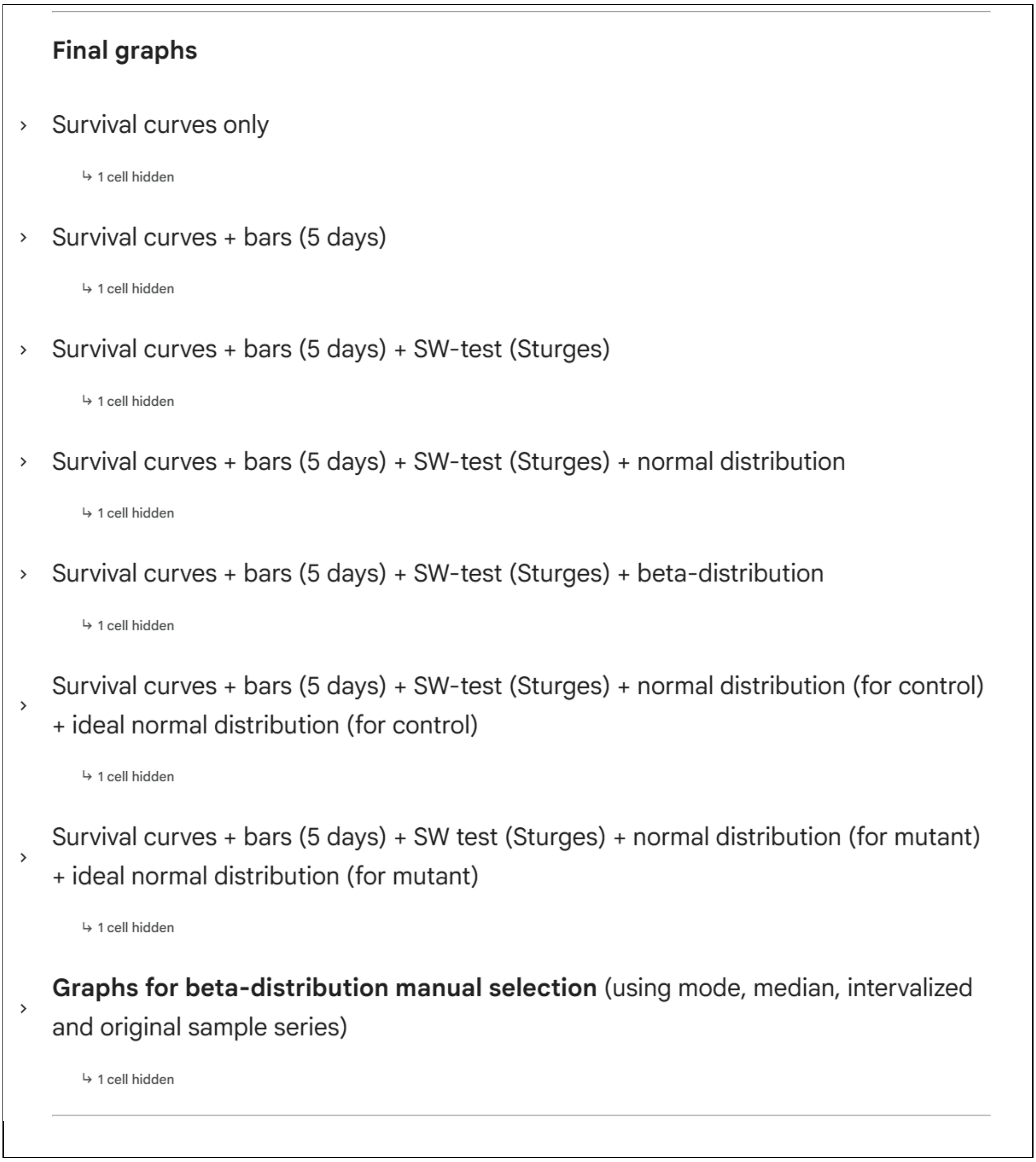

Figure. Cells for *.png files are presented.

ADVANCED SETTINGS

You can change the method of graph generation using the ‘**Advanced settings**’ cell.

The graph plotting method is controlled by a nested dictionary of settings, comprising three sections: ‘show’, ‘lines’ and ‘methods’. The base configuration is specified by the ‘base_config’ function.

The figure below shows the base configuration.

```
def base_config():
    return {
        "show": {
            "bars": True,
            "sub": True,
            "legend": True,
        },
        "lines": {
            "type": None
        },
        "methods": {
            "mod_or_median": ["TREE", "TREE"],
            "Intervalisation_mode": ["AUTO", "AUTO"],
            "Initial_day": [0, 0],
            "Ideal_curve_line": [0, 0],
            "beta_step": (1/3)
        },
    }
```

Figure. Base configuration.

The settings can be altered by calling the 'make_config' function with the respective arguments in the following format:
make_config(section_name = {parameter_name : argument})

You can see the example in the figure below and the arguments in the tables on the next page.

The 'make_config' function copies the settings set in 'base_config' and applies the changes specified in the brackets to them.

Each configuration mode must be assigned its own variable. These variables must then be included in the 'parameters' array, separated by commas.

ADVANCED SETTINGS for creating custom graphs

```
# MANUAL MODE
mode1 = make_config(show={"sub": False, "bars": True})
mode2 = make_config(lines={"type": "beta"}, methods={"mod_or_median": ["MODE","MEDIAN"]})
parameters = [mode1, mode2]

# CUSTOM GRAPHS
for allele in genotype_names:
    for pair in SAMPLES:
        for parameter in parameters:
            print (allele, pair)
            draw_plot(allele, pair, ls_data, raw_data, parameter)
```

Figure. An example of a configuration entry. Each mode variable is defined by settings passed via ‘make_config’ and all the variables are included in the parameters array. This array will be used to build graphs.

The tables of available parameters and their arguments are given below.
The inner dictionary 'show' accepts values in the True/False format and allows you to hide certain parts of the graph.

Table. The parameters of the inner dictionary 'show'.

**make_config(show = {parameter : argument})**		
**Parameters**	**Arguments**	**Description**
‘bars’	True	Show the bars
	False	Hide the bars
‘sub’	True	Show the survival curves
	False	Hide the survival curves
‘legend’	True	Show the legend
	False	Hide the legend

The inner dictionary ‘curves’ controls which curves are displayed on the graph.

Table. The inner dictionary ‘curves’.

**make_config(curve = {parameter : argument})**		
**Parameters**	**Arguments**	**Description**
‘type’	‘beta’	Constructs β-distributions for control and mutant.

	'normal'	Constructs normal distributions for control and mutant.
	'ideal'	Constructs normal distribution and ideal normal distribution, either for control or for mutant.
	None	No curves.

The inner dictionary 'methods' controls the parameters controls the parameters for calculating the specific curves.

Table. The inner dictionary 'methods'.

**make_config(methods = {parameter : argument})**		
**Parameters**	**Arguments**	**Description**
'Intervalisation_mode'	'INT'	Calculation of *p* and *q* using intervalised by 5 days data.
	'NONINT'	Calculation of *p* and *q* using non-intervalised (original) data.
	'AUTO'	Decide the type of data (intervalized/non-intervalized) automatically.
'mod_or_median'	'MODE'	Calculation of *p* and *q* using mode.
	'MEDIAN'	Calculation of *p* and *q* using median.
	'TREE'	Calculation of *p* and *q* using the decision tree.
	'BELL'	Returns $p = 3.5$, $q = 3.5$.
'Initial_day'*	[N, N], where each N is an integer within the range 0 to 4.	Used only for intervalised data. When 'Intervalisation_mode' is set to 'AUTO', the search for acceptable p and q values ($p \geq 1$ and $q \geq 1$) begins from set 'Initial_day'.<br><br>The first value is for the control cohort and the second is for the mutant cohort.
'Ideal_curve_line'	[N, N], where each N takes the value of either 0 or 1.	Used in generation of comparisons between normal and ideal normal distributions. Determines which sample from the pair will be used to generate each curve.<br><br>The first value in array determines the normal distribution curve (0 = control; 1 = mutant), and the second one determines the ideal normal distribution curve (0 = control; 1 = mutant).

‘beta_step’	Any number.	Step size for drawing the beta distribution; does not affect the calculation but does affect the smoothness of the curve. The smaller the step size, the more points on the graph will be used to plot the beta distribution.<br><br>For example, 'beta_step' = 1/2 means that a point for the curve is calculated for each half of the day on the graph; 'beta_step' = 1/3 means every one-third; and so on.

* In the case of a *β*-distribution construction (calculation of the Mode and Median followed by calculation of *p* and *q*) on intervalized data (as required according to the decision tree in Fig. S9), we sum all deaths within each interval for intervalization, e.g. [3, 3, 3, 4; 6, 7, 7, 7, 8, 9; etc.], where each number represents the lifespan of the individual and the semicolons formally represent the boundaries of the intervals. As a result, we obtain a series consisting of the number of deaths in each interval, e.g. [4, 6, etc.]. This series is then converted into a series of pseudo-lifespans, in the form [0, 0, 0, 0; 5, 5, 5, 5, 5, 5], where the semicolons mark the boundaries of the intervals, and the numbers indicate the pseudo-lifespans of the individuals. This series state is called 'Initial_day':[0]. If 'Initial_day':[1] is used, the series takes the form [1, 1, 1, 1; 6, 6, 6, 6, 6, 6] and etc. The 'Initial_day' value can be set from 0 to 4, since intervalization occurs every five days (five days in a column). Thus, we assign a day of death (pseudo-lifespan) to each individual in the interval in order to calculate the Mode and Median for the intervalized lifespan series.

The code in the '**ADVANCED SETTINGS**' cell is initially commented out to avoid unnecessary errors. To generate modified graphs, the code must be uncommented.

To uncomment it, open the cell and click anywhere inside the block of code. Then press Ctrl+A, followed by Ctrl+/. The cell is now uncommented and you can enter the settings (Figure below). Once you have entered the desired settings, you can run the code, the specified graphs will appear as the cell's output.

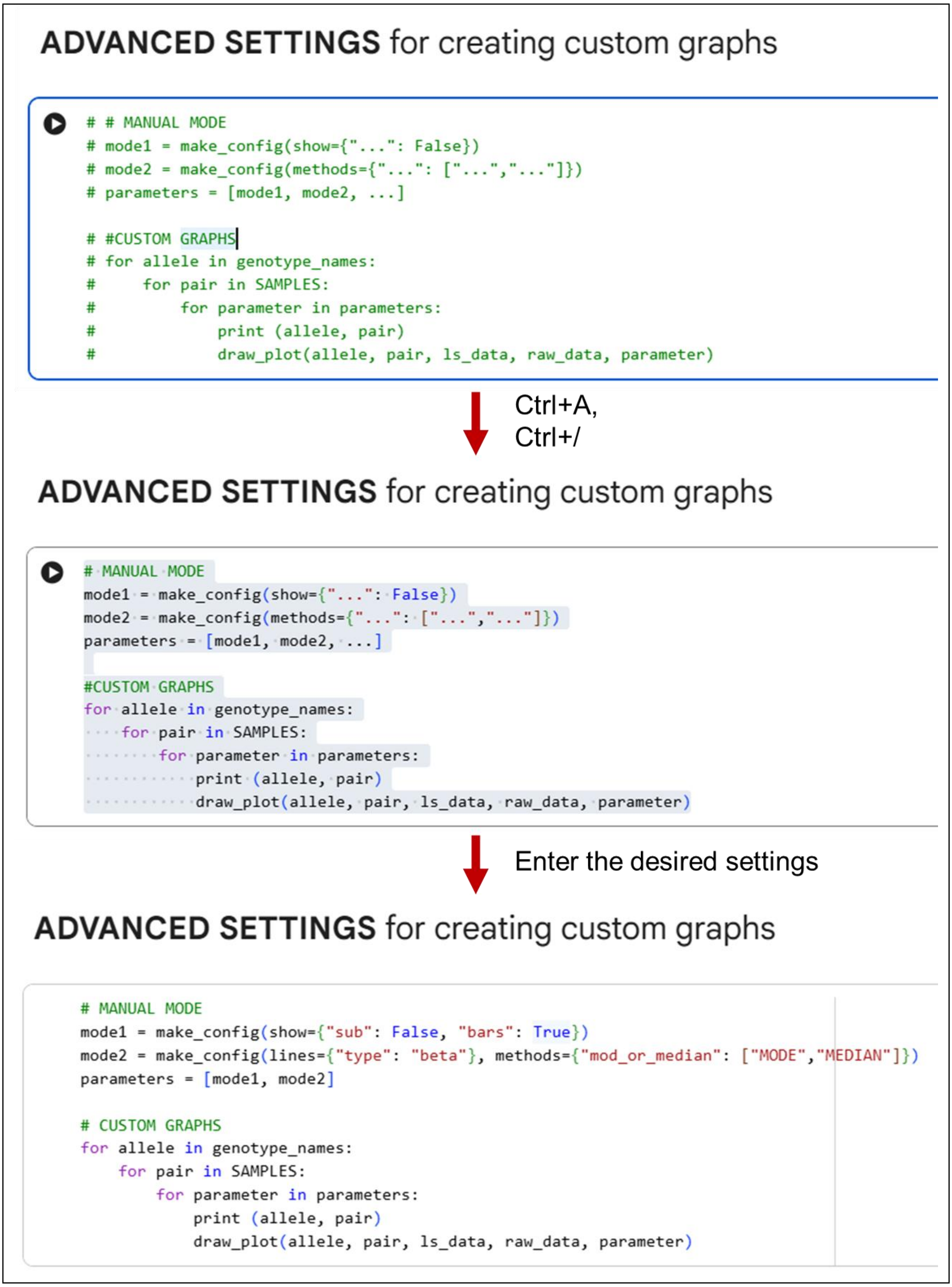

Figure. Uncommenting and entering user settings.

Supplementary S5

Calculation of the two-sample Kolmogorov-Smirnov test.

See list Appendix #5 in MS Excel supplementary file.

References

Feller, W. (1948). On the Kolmogorov-Smirnov Limit Theorems for Empirical Distributions. *The Annals of Mathematical Statistics* 19, 177–189. doi: 10.1214/aoms/1177730243

Kolmogoroff, A. (1933). “Sulla Determinazione Empirica di una Legge di Distribuzione.” *”Giornale dell’ Istituto Italiano degli Attuari.* 4, 83–91.

Massey, F. J. (1951). The Kolmogorov-Smirnov Test for Goodness of Fit. *Journal of the American Statistical Association* 46, 68–78. doi: 10.1080/01621459.1951.10500769

Smirnov, N. (1948). Table for Estimating the Goodness of Fit of Empirical Distributions. *The Annals of Mathematical Statistics* 19, 2, 279–281. doi: 10.1214/aoms/1177730256
